# Shared as well as distinct roles of EHD proteins revealed by biochemical and functional comparisons in mammalian cells and *C. elegans*

**DOI:** 10.1186/1471-2121-8-3

**Published:** 2007-01-18

**Authors:** Manju George, GuoGuang Ying, Mark A Rainey, Aharon Solomon, Pankit T Parikh, Qingshen Gao, Vimla Band, Hamid Band

**Affiliations:** 1 Division of Molecular Oncology, Evanston Northwestern Healthcare Research Institute, Department of Medicine, Feinberg School of Medicine, Northwestern University, Evanston, Illinois, USA; 2 Division of Cancer Biology, Evanston Northwestern Healthcare Research Institute, Department of Medicine, Feinberg School of Medicine, Northwestern University, Evanston, Illinois, USA; 3 Department of Biochemistry, Molecular Biology and Cell Biology, Northwestern University, Evanston, Illinois, USA; 4 Robert H. Lurie Comprehensive Cancer Center, Northwestern University, Evanston, Illinois, USA

## Abstract

**Background:**

The four highly homologous human EHD proteins (EHD1-4) form a distinct subfamily of the Eps15 homology domain-containing protein family and are thought to regulate endocytic recycling. Certain members of this family have been studied in different cellular contexts; however, a lack of concurrent analyses of all four proteins has impeded an appreciation of their redundant versus distinct functions.

**Results:**

Here, we analyzed the four EHD proteins both in mammalian cells and in a cross-species complementation assay using a *C. elegans *mutant lacking the EHD ortholog RME-1. We show that all human EHD proteins rescue the vacuolated intestinal phenotype of *C. elegans rme-1 *mutant, are simultaneously expressed in a panel of mammalian cell lines and tissues tested, and variably homo- and hetero-oligomerize and colocalize with each other and Rab11, a recycling endosome marker. Small interfering RNA (siRNA) knock-down of EHD1, 2 and 4, and expression of dominant-negative EH domain deletion mutants showed that loss of EHD1 and 3 (and to a lesser extent EHD4) but not EHD2 function retarded transferrin exit from the endocytic recycling compartment. EH domain deletion mutants of EHD1 and 3 but not 2 or 4, induced a striking perinuclear clustering of co-transfected Rab11. Knock-down analyses indicated that EHD1 and 2 regulate the exit of cargo from the recycling endosome while EHD4, similar to that reported for EHD3 (Naslavsky *et al*. (2006) Mol. Biol. Cell 17, 163), regulates transport from the early endosome to the recycling endosome.

**Conclusion:**

Altogether, our studies suggest that concurrently expressed human EHD proteins perform shared as well as discrete functions in the endocytic recycling pathway and lay a foundation for future studies to identify and characterize the molecular pathways involved.

## Background

Endocytosis is an essential cellular process that regulates the delivery of specific cargo and lipid membranes to appropriate subcellular destinations [1]. Endocytic traffic of signaling receptors into lysosomal versus recycling endosomal pathways also provides a fundamental mechanism to control cellular responses to environmental changes. Finally, the endocytic pathway intersects other intracellular transport pathways such as the secretory pathway. Understanding the molecular basis of regulated transport within the endocytic pathway is, therefore, of broad interest and substantial biological significance.

Protein-protein interactions provide a central mechanism to control cellular functions, and regulatory proteins within a given functional pathway are often characterized by the presence of related modular protein-protein interaction domains. A large subset of proteins involved in the regulation of endocytic trafficking events contain an Eps15 Homology (EH)^1 ^domain, first identified as three repeated copies in the epidermal growth factor receptor pathway substrate 15 (Eps15) [2,3]. There are over 50 EH domain-containing proteins known [4] and many of them, such as Eps15, Eps15R, intersectin, POB1, END3 and REPS1, are involved in the early stages of endocytosis [5]. EH domains recognize an Asn-Pro-Phe (NPF) motif within target proteins to assemble protein complexes that function at various steps during endocytic traffic, such as cargo selection and formation of clathrin-coated pits at the plasma membrane [6].

The recently identified subfamily of EH domain-containing proteins (EHD1-4) are characterized by a unique domain organization distinct from other EH domain-bearing proteins: they contain a single EH domain at the C-terminus, a central coiled-coil region and a phosphate-binding loop (P-loop) capable of binding nucleotides in the N-terminal region [7-9]. The genes encoding these proteins are located on different chromosomes, EHD1 on 11q13 [7], EHD2 on 19q13.3, EHD3 on 2p21 and EHD4 on 15q11.1, yet they share a high degree of homology at the nucleotide and amino acid levels [8]. The presence of four EHD proteins in mammals appears to reflect recent duplications as a single gene is found in non-mammalian organisms [8].

The presence of an EH domain suggests a possible role for these proteins in receptor-mediated endocytosis. Consistent with this idea, mutations in receptor-mediated endocytosis-1 (RME-1, the only *C. elegans *homolog) led to defective endocytic traffic of the yolk protein receptor RME-2 as well as aberrant trafficking in intestinal cells leading to a vacuolated intestine phenotype in *C. elegans *[10]. RME-1 is most closely related to human EHD1 [10] and it has been shown that EHD1 is involved in the recycling of a variety of receptors from the endocytic recycling compartment (ERC) to the plasma membrane [11-14]. Previous studies have also shown that EHD1 and 3 reside in the ERC as indicated by their colocalization with transferrin [11,15]. Furthermore, perturbations of EHD1 and 3 led to altered distribution and function of the ERC [11,16]. Other EHD proteins have been studied in specialized cells such as adipocytes and pheochromocytoma cells. EHD2 was isolated from GLUT4-enriched fractions of adipocytes and shown to regulate insulin-mediated translocation of GLUT4 to the plasma membrane [17,18]. EHD4, also called Pincher, has been shown to regulate neurotrophin receptor TrkA endocytosis in pheochromocytoma (PC12) cells [19].

Several EHD-binding proteins have been identified recently such as Rabenosyn-5 [12], SNAP29/GS32 [20,21], syndapin I and II [20,22], α-adaptin subunit of AP2 [21], reticulon (in the case of RME-1) [23], Rab11-FIP2 [16], EHBP1 [17,24] and Numb [25]; many of these contain one or more NPF motifs [26]. Biochemical studies and yeast two-hybrid experiments have shown that mutations in the P-loop and coiled-coil region interfere with oligomerization of RME-1/EHD1 [9] and EHD3 [16]. Despite the emerging information on their role in endocytic recycling of different receptors and identification of their interaction partners, little is known about EHD proteins and how they function in cells.

While these initial studies have begun to establish the role of EHD proteins in endocytic traffic, in particular at the ERC, there has been a distinct lack of biochemical and functional comparisons of mammalian EHD proteins in a single cellular system. As suggested by Naslavsky and Caplan, the overall identity of EHD proteins is slightly higher than that of their EH domains [27] and the comparison of all human EHD proteins in parallel could, therefore, provide important clues to their function in mammalian cells. Here, we have compared the four human EHD proteins with respect to their expression, localization and function to identify their distinct and shared cellular functions. These studies, together with recent comparison of EHD1 and 3 by the Caplan group [16], should serve as a basis for further efforts to understand the molecular basis of EHD protein function.

## Results

The four mammalian EHD proteins are highly related in sequence as well as domain structure [8,27]. However, it is not clear whether they have distinct roles in mammalian cells, perform similar function in different cellular contexts or are, to some degree, redundant in function. To begin to address these issues, we have carried out simultaneous biochemical and functional analyses of human EHD1-4 both through reconstitution of function in the *C. elegans *mutant *rme-1*(*b1045*), in which the single EHD ortholog RME-1 is mutated, and by expression in mammalian cells.

### All human EHD proteins rescue the defective rme-1 function in the intestine of *C. elegans rme-1*(*b1045*)

The role of EHD proteins in endocytic recycling was first revealed by the isolation of *C. elegans *EHD ortholog RME-1 as a regulator of endocytosis [10,11]. Among the various defects, *rme-1 *mutants were shown to develop large vacuoles in the intestine at the larval L4 stage which increased in number with age. These vacuoles accumulate fluid phase markers within minutes of application to the basolateral surface, but not when applied to the apical surface, indicating that they arise from a specific basolateral trafficking defect [28]. RME-1 is most closely related to mammalian EHD1, and GFP-RME-1 and -EHD1 formed similar vesicular structures and colocalized with transferrin in Chinese Hamster Ovary (CHO) cells [11]. Given the overall conservation among mammalian EHD proteins, we used mutant *C. elegans *with anon-functional EHD ortholog in a cross-species complementation strategy to test if all or selected human EHD proteins function to regulate endocytic trafficking.

**Figure 1:**
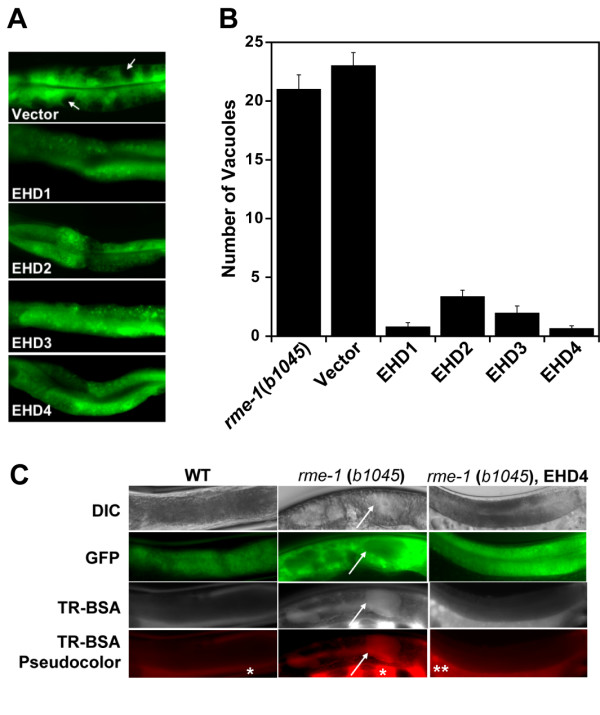
**All human EHD proteins rescue the vacuolated intestinal phenotype in the intestine of *C. elegans rme-1 *(*b1045*)**. (A) Micrograph images of middle intestinal regions of transgenic animals expressing the human EHD proteins. The *rme-1*(*b1045*) worms were injected with pVha-6::SL2-GFP (50 ng/μl) or with the same construct containing the human EHD cDNAs along with myo2::GFP (100 ng/μl) as a co-injection marker. Intestinal vacuoles are viewed as spaces devoid of green fluorescence in the *rme-1*(*b1045*) mutant (arrows). (B) Intestinal vacuoles were counted in at least 3 independent transgenic lines expressing no vector (*rme-1*(*b1045*)), vector alone (Vector), or a vector containing EHD1-4. (C) Basolateral endocytosis assay of the intestinal vacuoles. Adult hermaphrodites were microinjected with 1 mg/mL Texas-Red BSA (TR-BSA) into the pseudocoelom and examined for uptake in intestinal vacuoles. Lack of accumulation of TRed-BSA microinjected into the pseoudocelum in wild-type (WT) worms (N2 Bristol strain) (left). Rapid accumulation of TR-BSA in the enlarged intestinal vacuoles (arrows) in the *rme-1*(*b1045*) mutant worms (middle). *rme-1*(*b1045*) worms rescued with human EHD4 do not display accumulation of the dye in any intestinal cells (right) similar to WT animals. * – pseudocoelom, ** – gonads. DIC – differential interference contrast microscopy.

Since the deletion mutant allele *rme-1*(*b1045*) (deletion of entire coding region from codon 240 onwards) was shown to lack any detectable RME-1 protein and did not appear to be dominant-negative [10], we chose this allele for our rescue experiments. The vacuolated intestinal phenotype is a suitable assay system since it is easy to score and has been successfully used to identify a novel gene involved in endocytic recycling in intestinal cells [29]. The human EHD cDNAs were cloned into an expression vector driven by the intestine-specific *Vha-6 *promoter [30] with an IRES sequence separating the EHD gene from the coding sequence of a GFP marker. This allowed easy scoring of the vacuoles as circular regions devoid of green fluorescence (Figure 1A, arrows). Similar to the *rme-1*(*b1045*) animals, which had an average of 21 ± 1.2 vacuoles per animal, GFP-expressing transgenic *rme-1*(*b1045*) animals (vector) had an average of 23 ± 1.3 vacuoles per animal (Figure 1B). Wild type (N2 Bristol) animals never developed intestinal vacuoles. As expected from the close structural similarity with RME-1, expression of human EHD1 was able to rescue the vacuolated intestinal phenotype comparable to that observed with *p*rme-1::GFP-RME-1 construct (0.8 ± 0.4 vacuoles per animal, Figure 1B) [10]. Notably, we observed that the other human EHD proteins could also rescue the intestinal phenotype to an extent comparable to that seen with EHD1 (Figure 1B). To further examine the rescue of the basolateral trafficking defect, we injected the fluid phase marker Texas Red-BSA (TR-BSA) into the psuedocoelom. The TR-BSA accumulated within one minute of injection in the intestinal vacuoles of *rme-1*(*b1045*) animals (Figure 1C, left). In wild type animals, no accumulation of TR-BSA was observed reflecting the efficient recycling back to the pseudocoelom of the basolaterally endocytosed fluid phase markers (Figure 1C, middle) [28]. As expected, rescued *rme-1*(*b1045*) animals did not accumulate any TR-BSA similar to wild type animals, indicating that the basolateral trafficking defect was rescued in these worms (Figure 1C, right). When TR-BSA was presented via the apical surface using feeding, it did not accumulate in the vacuoles in either the mutant or the rescued worms indicating that apical endocytosis and recycling were unaffected in these worms (data not shown). Collectively, these experiments indicate that human EHD proteins retain the basic function of their *C. elegans *ortholog to control endocytic trafficking.

### All human EHD proteins are concurrently expressed in multiple cultured cell lines and several tissues

**Figure 2:**
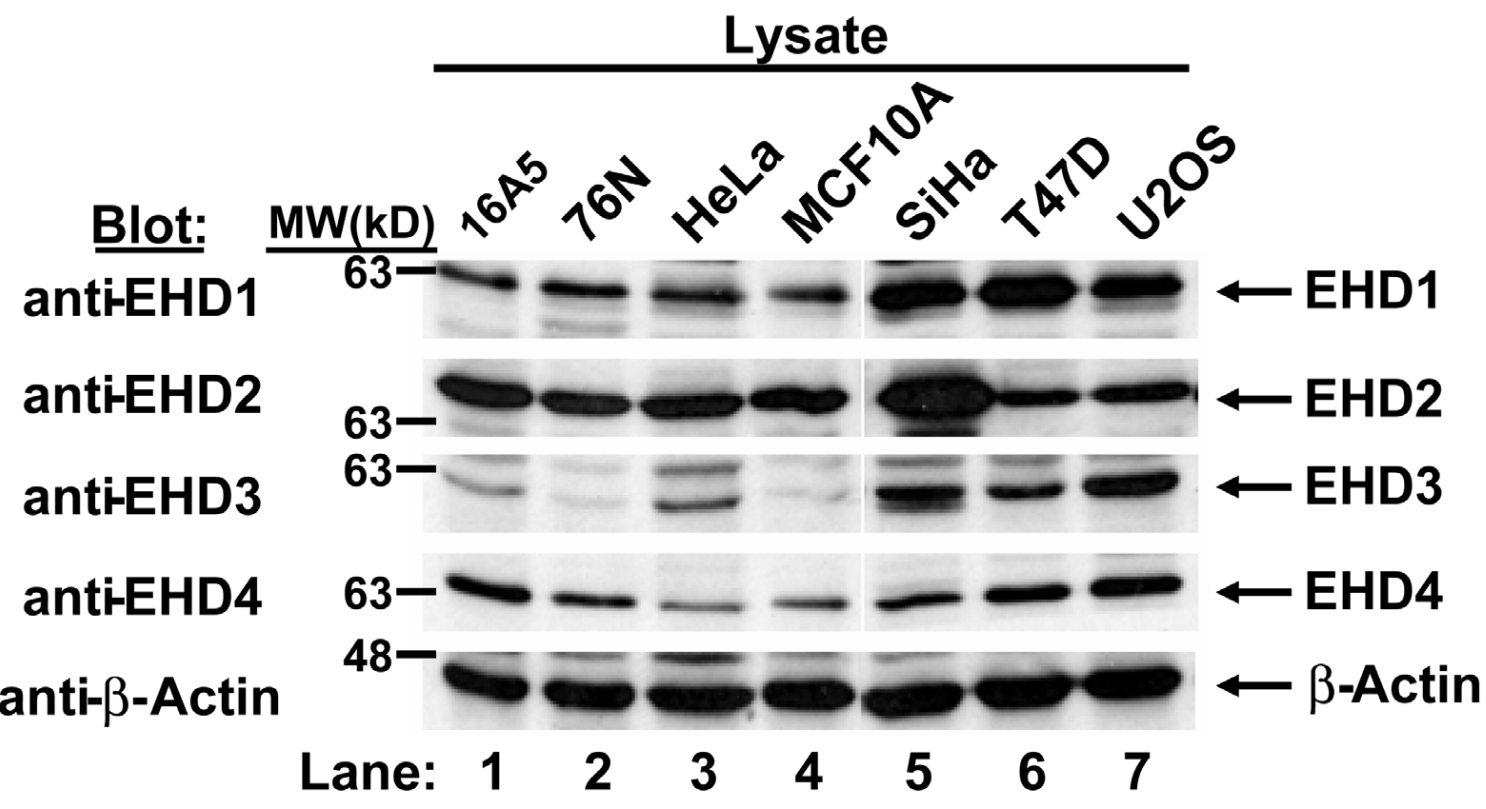
**All human EHD proteins are concurrently expressed in multiple cell lines**. Aliquots of 100 μg cell lysates were resolved by 8% SDS-PAGE and subjected to immunoblotting with rabbit anti-peptide antisera raised against specific EHD proteins. Endogenous EHD proteins were detected in each cell lysate. The specificity of the antisera for EHD proteins is shown in Additional File 1 using GFP-fusion proteins. Relative molecular weight (MW) markers are indicated in kiloDaltons (kD). As a loading control, β-Actin was blotted.

**Figure 3:**
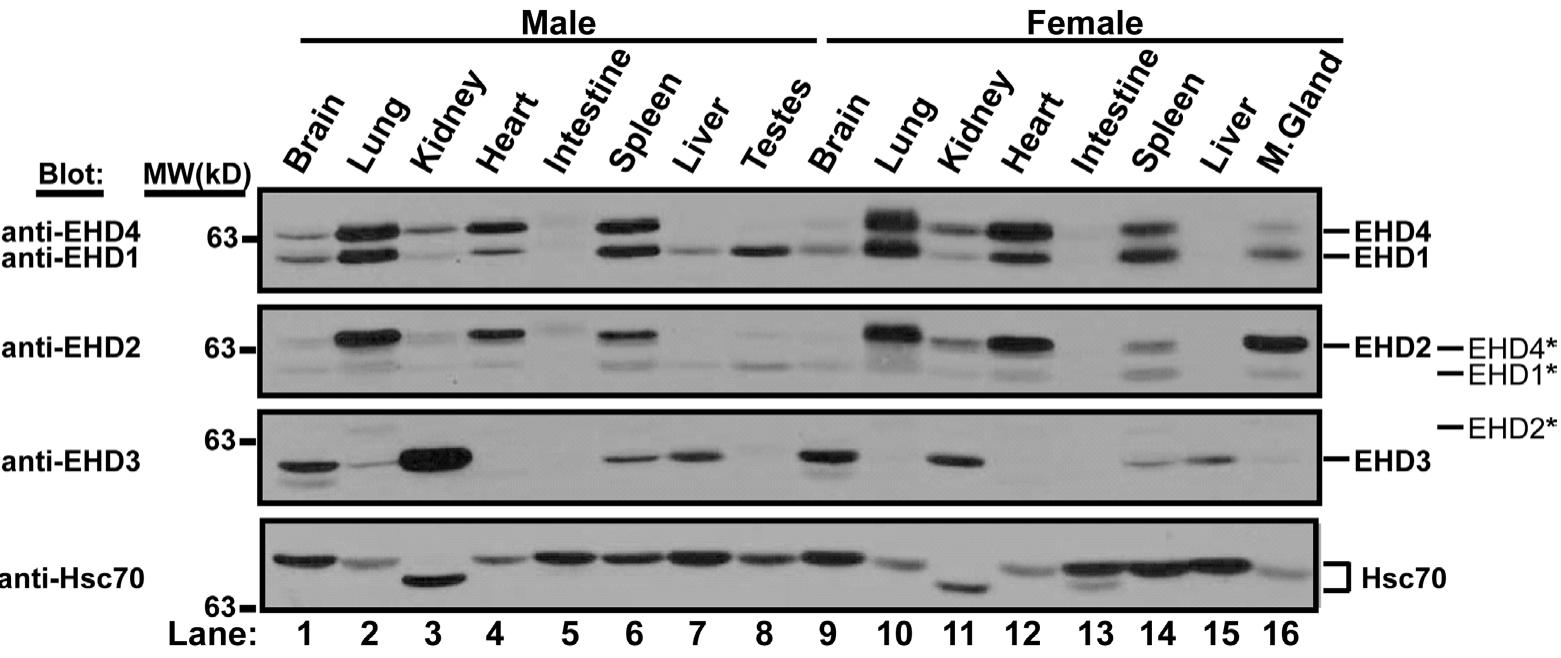
**EHD protein expression in normal mouse tissues**. An 18 week-old male and female C57BL/6 mouse was sacrificed, organs were removed and lysed in tissue lysis buffer as described in Methods. Aliquots of 100 μg tissue lysate were separated using 8% SDS-PAGE and a Western blot was performed using antibodies raised against human EHD proteins. The membrane was serially stripped and reprobed beginning with both EHD1 and EHD4, followed by EHD2, EHD3 and Hsc70 antibodies. The * denotes bands that bled through from the previous blot following stripping. Differential mobility of Hsc70 may represent tissue specific isoforms. Blots shown have exposure times of less than 10 seconds, upon longer exposures, most EHD proteins can be seen in each organ shown. Relative molecular weight (MW) markers are indicated in kiloDaltons (kD). As a loading control, Hsc70 was blotted. M. gland – mammary gland.

Rescue of defective endocytic recycling in mutant worms by all human EHD proteins strongly suggested that these proteins might participate in regulation of the endocytic pathway in mammalian cells. The emergence of multiple RME-1 orthologs (EHD1-4) in higher mammalian organisms could reflect tissue-specific expression or further functional diversification as a result of distinct localization or interaction with specific protein partners. Therefore, we checked whether all EHD proteins or only selected members are expressed concurrently in a given mammalian cell. Previously, EHD-encoding mRNAs were detected in various human tissues [7,8], but concurrent endogenous EHD protein expression has only been recently shown for HeLa cells and suggested for normal mouse fibroblasts [16]. To assess endogenous EHD protein expression in a broader array of human cell lines, we developed and characterized rabbit anti-peptide antisera that specifically recognize individual human EHD proteins based on selective immunoblotting of exogenously expressed EHD-GFP proteins in HEK 293T cells (Additional File 1). Western blots of endogenous proteins using these antibodies demonstrated that each of the 7 selected human cell lines expressed all four EHD proteins (Figure 2). Analysis of nearly fifteen additional cell lines confirmed this general conclusion (data not shown). Endogenous EHD proteins were also detected in mouse tissue extracts using the same antibodies (Figure 3). All EHD proteins are expressed in most tissues tested, although their levels varied between tissues. For example, EHD3 is highly expressed in kidney and brain, whereas, EHD1, 2 and 4 are highly expressed in lung, heart and spleen. Tissue-specific levels of expression may point to differences in their functions.

### All human EHD proteins form homo- and hetero-oligomers but EHD2 is relatively deficient in hetero-oligomerization

**Figure 4:**
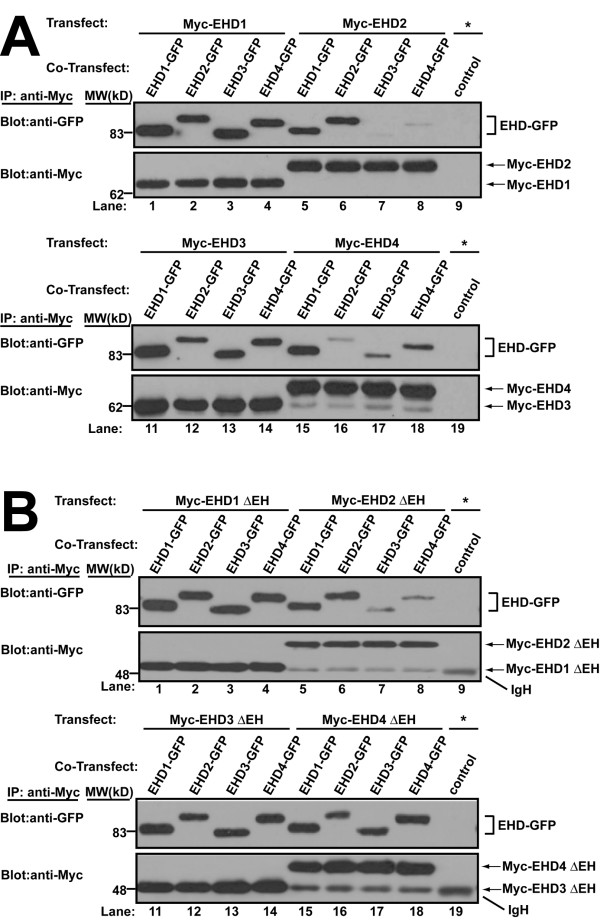
***In vivo *homo- and hetero-oligomer formation of human EHD proteins in mammalian cells**. (A) HEK 293T cells in 100-mm tissue culture dishes were co-transfected with DNA encoding a single Myc-EHD (2.5 μg) and one EHD-GFP (2.5 μg) construct. Cell lysates were prepared 26–30 h after transfection, rocked overnight at 4°C, and 1 mg aliquots of lysate were subjected to immunoprecipitation (IP) with 3 μg of anti-Myc (9E10) antibody followed by serial anti-Myc and anti-GFP immunoblotting. A negative control (control) using 3 μg of anti-Cbl-b mouse monoclonal IgG1 (lane 9 and 19) was carried out using the lysates transfected (*) as in lane 1 and lane 11, respectively. Further negative controls for the specificity of the co-IP are shown in Additional File 2C. The identity of the lower bands in lanes 15–18 of the anti-Myc blots are unknown. (B) HEK 293T were similarly co-transfected with DNA encoding a single Myc-EHD ΔEH (2.5 μg) and an EHD-GFP (2.5 μg) construct and IPs were carried out as above except that lysates were rocked only 1–2 hours at 4°C following lysis. This exception allowed positive detection of EHD2 ΔEH co-IPs whereas EHD1 ΔEH results were unaffected with further rocking. The identity of the lower bands of the anti-Myc blots in lanes 5–9 and 15–19 are the heavy chain of the mouse IgG (IgH) and are assumed to be masked by and comigrating with Myc-EHD1 ΔEH and Myc-EHD3 ΔEH in lanes 1–4 and 11–14, respectively. All blots are representative of 3 experiments and the lysates used for these IPs are shown in Additional File 2A–2B. Relative molecular weight (MW) markers are indicated in kiloDaltons (kD).

EHD proteins contain coiled-coil domains thought to be involved in oligomerization. Yeast two-hybrid interaction and mammalian cell co-expression studies have demonstrated the existence of RME-1/EHD1 homo-oligomers and EHD1/3 hetero-oligomers [9,15,16]. A functional P-loop and coiled-coil region are required for oligomerization and are essential for RME-1 and EHD1 function [9,16]. No such information is available for EHD2 and EHD4. One possible means for functional heterogeneity among EHD proteins could be their differential ability to form homo- and hetero-oligomers with other EHD proteins in a given cell. In order to assess the ability of human EHD proteins to form homo- and hetero-oligomers, we co-transfected Myc- and GFP-tagged EHD proteins in HEK 293T cells and assessed the presence of the EHD-GFP partner in anti-Myc immunoprecipitates. All EHD proteins formed homo-oligomers when GFP- and Myc-tagged forms of the same EHD protein were co-expressed (Figure 4A). Furthermore, EHD1 co-immunoprecipitated with each of the other three EHD proteins both when Myc-EHD1 was co-expressed with GFP-tagged EHD2, 3 or 4, or when EHD1-GFP was co-expressed with Myc-tagged EHD2, 3 or 4. Co-immunoprecipitation (co-IP) of Myc-EHD4 with GFP-tagged form of itself or EHD1 was always robust, whereas its co-IP with EHD2- or 3-GFP was always detectable but varied in extent. The co-IP of EHD2 with EHD3 or 4 proteins was substantially lower when compared to other oligomeric combinations, indicating that EHD2 may primarily exist as either a homo-oligomer or a hetero-oligomer with EHD1 or that it may be present in a different compartment. Differential co-IP was not due to differences in the expression of proteins as revealed by immunoblotting of whole cell lysates (Additional File 2A, B). Similar results were obtained using HeLa cells (data not shown). The general pattern of co-IP in homo- and hetero-oligomeric combinations was similar when Myc-tagged EH domain deletion (ΔEH) mutants of EHD proteins were co-expressed with full-length GFP-tagged proteins (Figure 4B) indicating that the oligomerization of the human EHD proteins are independent of their EH domain.

That co-IPs were not due to non-specific interactions of overexpressed proteins was demonstrated by the lack of co-immuniprecipitation of GFP-myotubularian-related protein 3 (MTMR3) when co-transfected with Myc-EHD1 and by the lack of co-IP of Myc-sorting nexin-1 (SNX1) co-transfected with EHD1-GFP (Additional File 2C). When the primary sequences of human EHD proteins were analyzed using COILS (a coiled-coil prediction software), we observed that amino acids 195–228 of EHD1 and 3 showed a strong tendency to form coiled-coils, with lower scores for EHD2 and 4 (Additional File 3). This is in general agreement with our co-IP results. Thus, while all human EHD proteins are capable of homo- and hetero-oligomerization, they differ in their choice of oligomerization partners within the same cellular milieu with preference for certain homo- and hetero-oligomeric combinations. Here, we show for the first time using co-IP analyses that all overexpressed human EHD proteins form homo- and hetero-oligomers with one another in mammalian cells. While endogenous co-IPs from cell lysates have not been successful thus far (data not shown), possibly due to uneven co-expression, our initial analyses of mouse tissue extracts using anti-human EHD1 antibodies indeed show co-IP of EHD1 with EHD2 or EHD4 (data not shown) supporting the likelihood that endogenous EHD proteins do oligomerize.

### Human EHD-GFP proteins localize to tubulovesicular structures in HeLa cells

**Figure 5:**
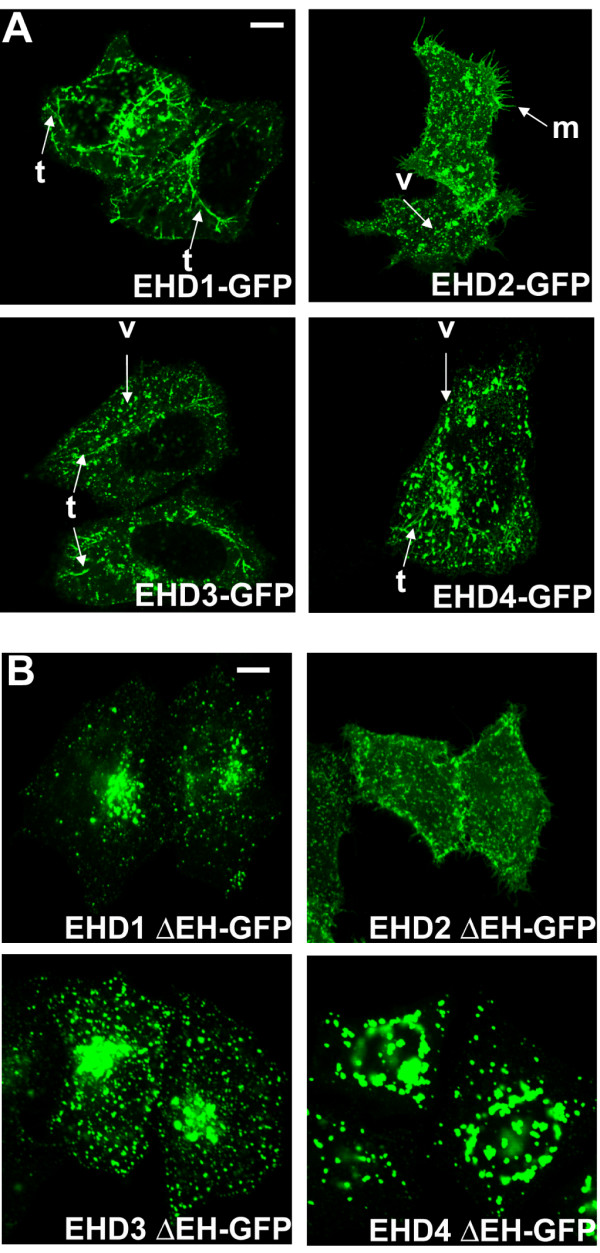
**Differential subcellular localization patterns of human EHD proteins**. (A) HeLa cells were transfected with C-terminal GFP-tagged EHD proteins for 24 h, fixed, mounted and scanned by a confocal microscope equipped with a 100× objective lens. Human EHD1-, 3- or 4-GFP are localized on tubular and vesicular structures in the perinuclear area, while EHD2-GFP is seen only in vesicular structures. Cells expressing EHD2 show microspikes; t, tubule; v, vesicle; m, microspikes. (B) HeLa cells were transfected with C-terminal GFP-tagged EHD ΔEH mutants for 24 h, fixed, mounted and scanned by a confocal microscope equipped with a 100× objective lens. Bar, 10 μm.

Previous studies have shown that wild type human EHD1 and EHD3 as well as the *C. elegans *RME-1 protein expressed in mammalian cells localize to tubulovesicular endocytic structures that include the ERC, although localization to additional vesicular structures was also noted [13,15]. Notably, the endocytic localization was completely lost in the loss-of-function P-loop mutants [9-11,13]. Therefore, we compared the subcellular localization patterns of human EHD proteins within a single cellular background to assess any differences in their localization. When GFP-tagged human EHD proteins were transfected into HeLa cells, we observed that EHD1, 3, and 4 were present in pleomorphic tubulovesicular structures in the perinuclear area, and in some cells these structures extended toward the periphery (Figure 5A). All transfected cells showed vesicular structures, but tubular structures varied greatly in number between cells and were entirely absent in some cells. A movie of an EHD1-GFP-transfected cell shows that these structures are dynamic; this is true of other EHD transfected structures (Additional File 4). In contrast, EHD2-GFP lacked perinuclear tubular structures and showed punctate vesicular staining throughout the cytoplasm. In addition, EHD2 was also observed at the plasma membrane where it often displayed a pattern of microspikes (Figure 5A). Comparable differences between EHD2-GFP and other GFP-tagged EHD proteins were observed upon transfection of CHO cells and immortal human mammary epithelial cell line MCF10A (data not shown).

### EHD proteins variably colocalize with each other

**Figure 6:**
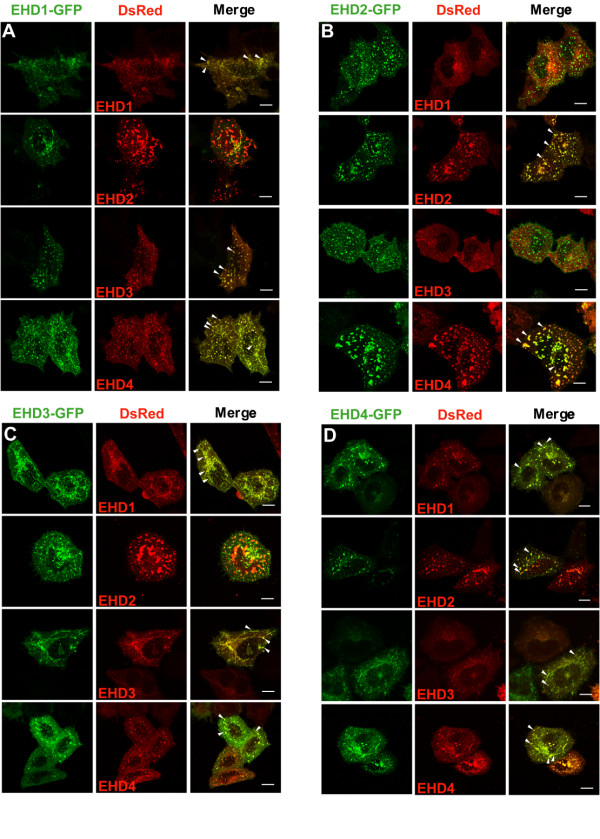
**Differential colocalization of GFP- and DsRed-tagged EHD proteins co-expressed in HeLa cells**. HeLa cells were co-transfected with C-terminal GFP- (green) and DsRed-tagged (red) EHD proteins for 24 h, fixed, mounted and scanned by a confocal microscope equipped with a 100× objective lens. Colocalization is indicated when similar shaped structures appear yellow in the Merge (arrowheads). (A) EHD1-GFP, (B) EHD2-GFP, (C) EHD3-GFP, (D) EHD4-GFP co-transfected with each EHD-DsRed construct. Bar, 10 μm.

We further assessed the colocalization of EHD proteins by co-expressing their GFP- and DsRed-tagged forms in HeLa cells. Comparison of GFP- versus the DsRed-tagged versions of each protein showed that different tags did not alter their overall patterns of localization, a conclusion further borne out by co-expressing the two tagged versions of the same protein (Figure 6). Importantly, EHD1 showed substantial colocalization with EHD3 and 4 and vice versa. In contrast, EHD2 showed little, if any, colocalization with EHD1 or 3 but showed partial colocalization with EHD4. Since the expression pattern of individual tagged EHD proteins did not appear to differ when expressed individually or with other EHD proteins (with the exception of the EHD2 and EHD4 pair), it appears unlikely that overexpression of one protein affects the expression pattern of the other. In the case of EHD4, we observed tubular and vesicular structures when it was overexpressed alone or with EHD1 and 3; however, when co-overexpressed with EHD2, EHD4 was primarily observed in larger vesicular structures.

Earlier studies with individual EHD proteins have shown an important functional role of the EH domain. Overexpression of EHD1 G429R, a mutation that is proposed to alter the conformation of the EH domain [10], in CHO and HeLa cells led to an altered appearance and function of the ERC apparently reflecting a dominant-negative phenotype of this mutant [11]. Therefore, we constructed GFP-tagged ΔEH mutants of each EHD protein, expressed them in HeLa cells and assessed their localization relative to the wild type versions. EHD1, 3 and 4 ΔEH-GFP mutants accumulated in prominent vesicular structures of varying sizes in the perinuclear region; these vesicular structures were quite large in cells expressing EHD4 ΔEH-GFP (Figure 5B). In contrast, the localization of the EHD2 ΔEH-GFP was similar to that of the wild type EHD2-GFP protein. These experiments complement our co-IP results and suggest that localization and oligomerization of EHD proteins may play an important role in their function.

### Effects of EHD protein expression on Rab11 localization

**Figure 7:**
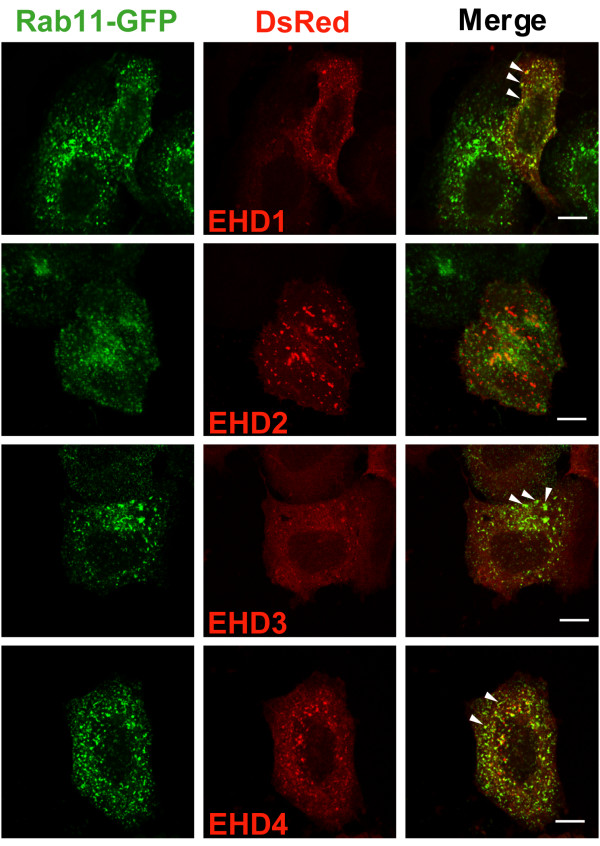
**Colocalization of EHD proteins with the endocytic recycling marker Rab11**. HeLa cells were co-transfected with Rab11-GFP (green) and EHD-DsRed (red) proteins for 24 h, fixed, mounted and scanned by a confocal microscope equipped with a 100× objective lens. Colocalization is indicated when similar shaped structures appear yellow in the Merge (arrowheads). Bar, 10 μm.

Small GTPases of the Rab family have characteristic cellular distributions and are known to regulate membrane traffic between different vesicular compartments [31] and some have been implicated in the control of trafficking within the endocytic pathway [32]. Of these, Rab11 is an important marker of the ERC as well as a regulator of transport through the ERC. Given the localization and apparent regulatory role of EHD proteins in the ERC [11], it appeared likely that these proteins may affect Rab11 function. We therefore assessed if EHD proteins colocalized with Rab4, 5, 7, 9 and 11. For this purpose, we co-expressed DsRed-tagged EHD proteins and GFP-tagged Rab proteins in HeLa cells. We observed that EHD1, 3 and 4 showed partial colocalization with Rab11 while EHD2 and Rab11 did not colocalize (Figure 7). We could not detect significant colocalization of EHD proteins with any of the other Rab proteins tested (data not shown). The incomplete colocalization of EHD1, 3 and 4 with Rab11 could reflect the dynamic state of vesicles on which these proteins colocalize, with rapid fusion and budding events allowing only partial colocalization at any given time point. Therefore, we used live cell imaging of HeLa cells co-transfected with EHD1-DsRed and Rab11-GFP to assess if Rab11 and EHD proteins show dynamic colocalization. Indeed, Rab11-positive (green) and EHD1-positive (red) vesicles were seen moving toward each other with transient coalescence of the green and red fluorescence (seen as yellow) followed by rapid return to green and red (Additional File 4). These results indicate that Rab11 and EHD1 colocalization reflects a dynamic state.

**Figure 8:**
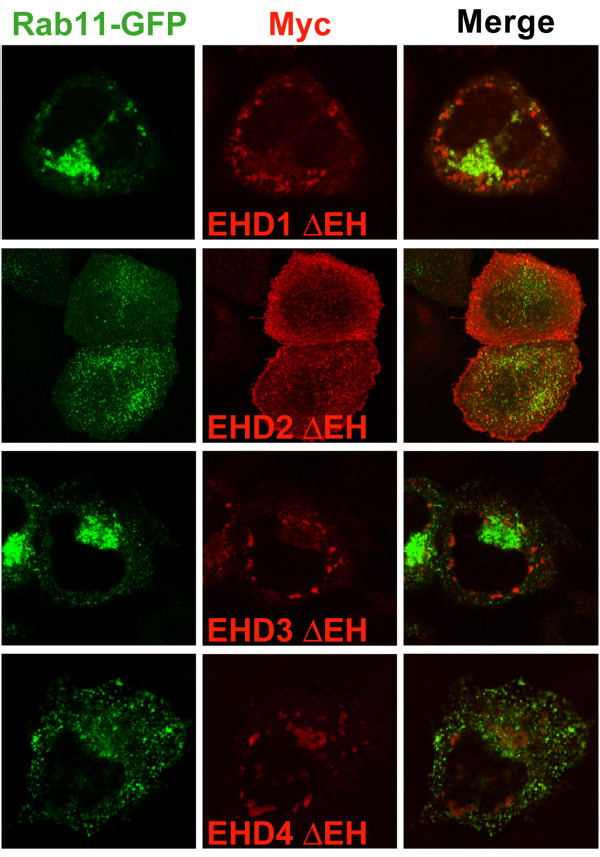
**Myc-EHD1 ΔEH and EHD3 ΔEH cause perinuclear clustering of Rab11-GFP**. HeLa cells were co-transfected with Myc-EHD ΔEH proteins (red) and Rab11-GFP (green) for 24 h, fixed, stained with antibodies for Myc (9E10), mounted and scanned by a confocal microscope equipped with a 100× objective lens.

We also examined the effect of expressing Myc-EHD ΔEH mutants in HeLa cells on Rab11-GFP localization. While none of the ΔEH mutants showed significant colocalization with Rab11-GFP, the expression of EHD1 and 3 ΔEH mutants resulted in a dramatic clustering of Rab11 in the perinuclear area (Figure 8) which was not seen when Rab11-GFP was co-transfected with wild type EHD proteins (Figure 7). In contrast, EHD2 and 4 ΔEH mutants did not produce noticeable alterations in Rab11 localization. Thus, it appears that some EHD proteins might act along with Rab11 in the same recycling pathway while others might not, raising the possibility that different EHD proteins may affect a common functional pathway through distinct mechanisms.

### Effect of EHD protein overexpression on transferrin trafficking

**Figure 9:**
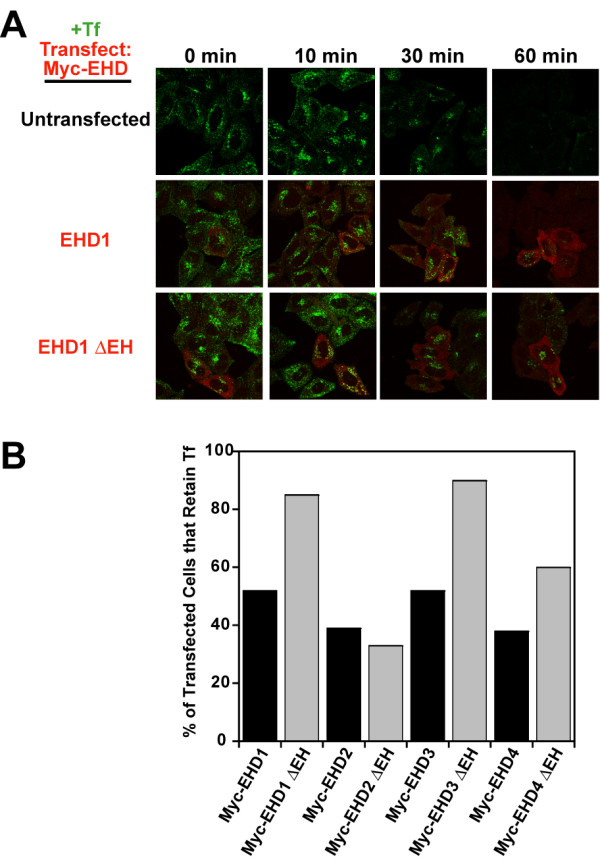
**Differential effects of wild type and ΔEH mutants of EHD1, 3, & 4 versus EHD2 on transferrin exit from the ERC**. Untransfected HeLa cells or cells transiently transfected with Myc-EHD1 or Myc-EHD1 ΔEH were loaded with Transferrin-coupled Alexa Fluor 488 (Tf, green) in internalization buffer at 37°C for 30 minutes 24 h after transfection, washed with ice-cold PBS and chased with serum-containing medium. The cells were fixed at various time points, stained with antibodies for Myc (9E10, red) and scanned on a confocal microscope. Similar experiments with EHD2-4 and EHD2-4 ΔEH are shown in Additional File 5. (B) Cells expressing Myc-EHD proteins (black bars) or Myc-EHD ΔEH proteins (grey bars) from a representative experiment were counted with respect to Tf retention after 60 min of chase. At least 200 cells were counted in each case.

Exit of internalized transferrin out of the ERC has been commonly used as an assay of ERC function [33-37]. Indeed, the first evidence for the role of EHD1 in mammalian endocytic transport employed the ability of EHD1 G429R mutant to delay transferrin exit from the ERC [11]. Several recent studies have addressed the role of EHD1, 2 and 3 in regulating endocytic recycling but the picture remains confusing due to the different transferrin receptor recycling assays used [11,12,16,17]. Therefore, we compared the effects of Myc-tagged wild type and Myc-EHD ΔEH proteins on transferrin recycling using a single assay [38] so that any differences could be ascribed to the proteins being studied rather than differences in methodology. HeLa cells transiently transfected with wild type or ΔEH mutants were allowed to internalize and accumulate fluorescently-labelled transferrin in the ERC and then chased with unlabeled transferrin for various time points. In untransfected cells, essentially no cells showed residual transferrin in the ERC at 60 min of chase (Figure 9A). Cells transfected with wild-type EHD proteins showed a retardation of transferrin exit from the ERC at 60 min of chase (Figure 9B, black). Furthermore, EHD1 and 3 ΔEH produced an even stronger effect compared to their wild type forms while the EHD4 mutant showed a milder effect (Figure 9B, grey). In contrast, the effect of EHD2 mutant was comparable to the wild type protein (Figure 9B). Thus, overexpression of wild type as well as ΔEH mutants appeared to perturb transferrin exit from the ERC. Notably, EHD2 behaved differently compared to other EHD proteins in this assay. In contrast to a previous report, we did not observe any defects of transferrin endocytosis in EHD2-transfected cells [17] (Additional File 5). However, as reported by Naslavsky *et al*., the block on transferrin exit in our studies was quite modest [16].

### Effect of siRNA-mediated knock-down of EHD proteins on transferrin recycling

**Figure 10:**
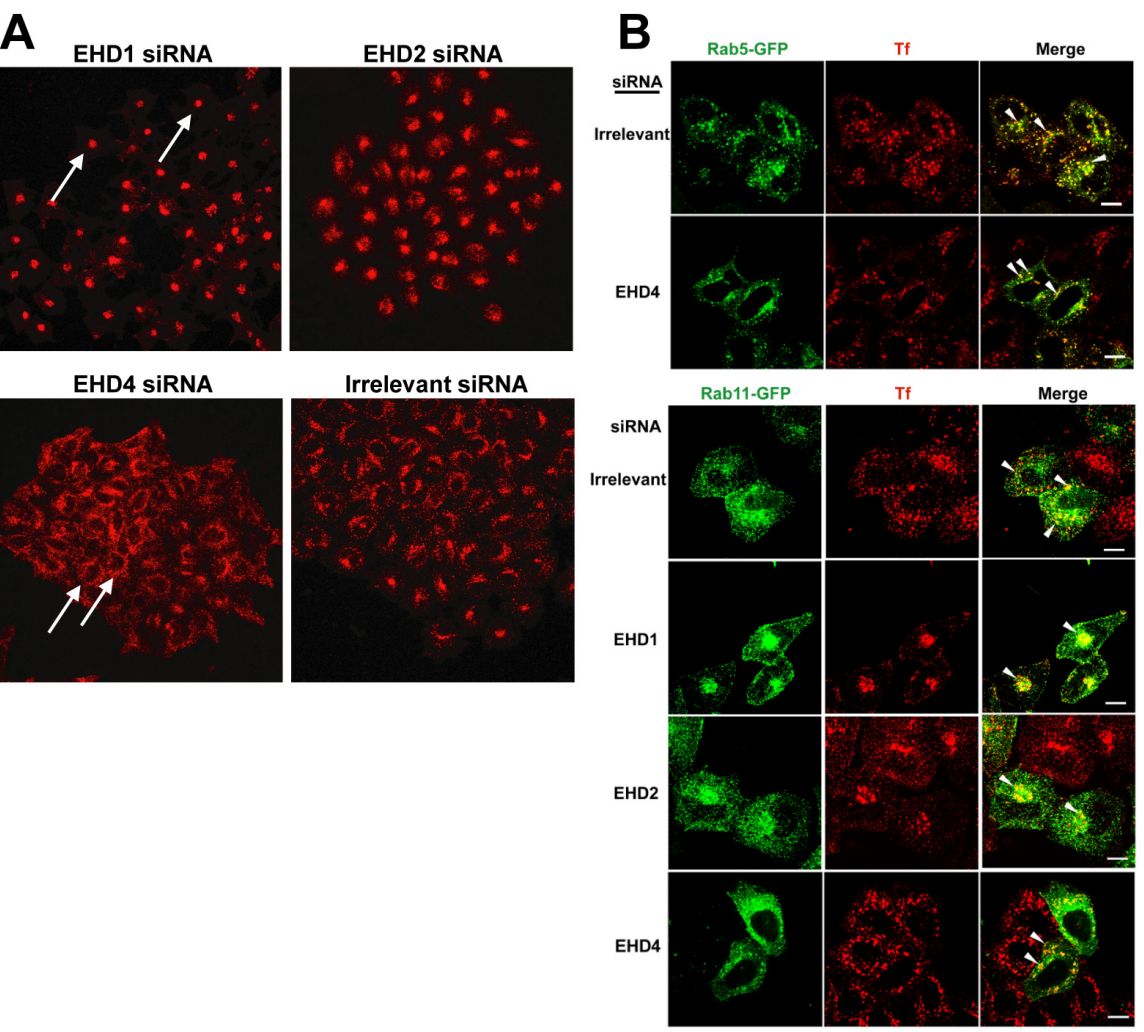
**siRNA-mediated EHD protein knock-down effects on transferrin loading in HeLa cells**. (A) HeLa cells were seeded on autoclaved glass coverslips in 6-well plates for 24 h followed by transfection of 200 pmol of double-stranded RNA oligonucleotides with irrelevant or EHD siRNA for 48 h prior to transferrin loading. Cells were starved for 30 min in starvation media followed by Transferrin-coupled Alexa Fluor 594 in internalization buffer at 37°C for 15 min, washed with ice-cold PBS, fixed and scanned using a confocal microscope equipped with a 40× objective lens. The arrows in the EHD1 siRNA depict an ERC transferrin loading phenotype while the arrows in the EHD4 siRNA depict an EE phenotype. (B) Cells were transfected with siRNA for EHD proteins for 24 h and further transfected with Rab5-GFP or Rab11-GFP for an additional 24 h. Cells were then loaded with labeled transferrin for 15 min as described in Methods. Arrowheads point to colocalized structures. Bar, 10 μm. Data are representative of 3 individual experiments.

In order to confirm the role of EHD proteins in transferrin recycling, we used siRNA-mediated knock-down of EHD proteins in HeLa cells and examined transferrin loading in these cells. The specificity of the EHD knock-down is shown in Additional File 6A. While EHD1, 2 and 4 siRNA resulted in a moderate reduction (> 40%) in protein levels (Additional File 6B), EHD3 knock-down using siRNA sequences from Naslavsky *et al*. [16] showed only a modest reduction in protein levels in our hands (~14%) but showed an identical transferrin phenotype as published for EHD3 (data not shown). As shown in Figure 10A, EHD1 siRNA caused the accumulation of transferrin-containing vesicles in the perinuclear area, in accordance with the observations made previously using identical siRNA sequences for EHD1 [16]. EHD2 siRNA induced a perinuclear transferrin accumulation phenotype similar to EHD1 siRNA, while EHD4 siRNA induced a peripheral transferrin accumulation phenotype, similar to the early endosome phenotype published for EHD3 knock-down [16] (Figure 10A).

Naslavasky *et al*. have previously reported the characterization of the compartment in which transferrin accumulates following EHD3 knock-down [16]. These investigators showed that early endosomal antigen 1 and Rab5 were absent from the perinuclear region in EHD3 knock-down cells and instead these markers colocalized with transferrin in enlarged peripheral early endosome (EE) structures [16]. We transfected EHD4 knock-down cells with Rab5-GFP and observed that Rab5-GFP was excluded from the perinuclear area and colocalized with transferrin in the periphery indicating that these are indeed early endosomes (Figure 10B). Further, we colocalized Rab11-GFP with the perinuclear vesicular structures that accumulate transferrin in cells with EHD1 and 2 knock-down, indicating that they are the ERC (Figure 10B). In addition, we observed that cells with EHD1 siRNA showed clustering of Rab11-GFP in the perinuclear area similar to cells overexpressing the EHD1 ΔEH mutant (Figure 8). Furthermore, confirming results published by the Caplan group, we noted that siRNA-mediated EHD3 knock-down resulted in the exclusion of Rab11 from the ERC (data not shown) [16]; notably, EHD4 siRNA had a similar effect on Rab11-GFP localization (Figure 10B). These experiments confirm that EHD1 and 2 knock-down result in the accumulation of transferrin in the ERC (designated the ERC phenotype) and EHD4 knock-down results in the accumulation of transferrin in the EE compartment (designated the EE phenotype).

**Figure 11:**
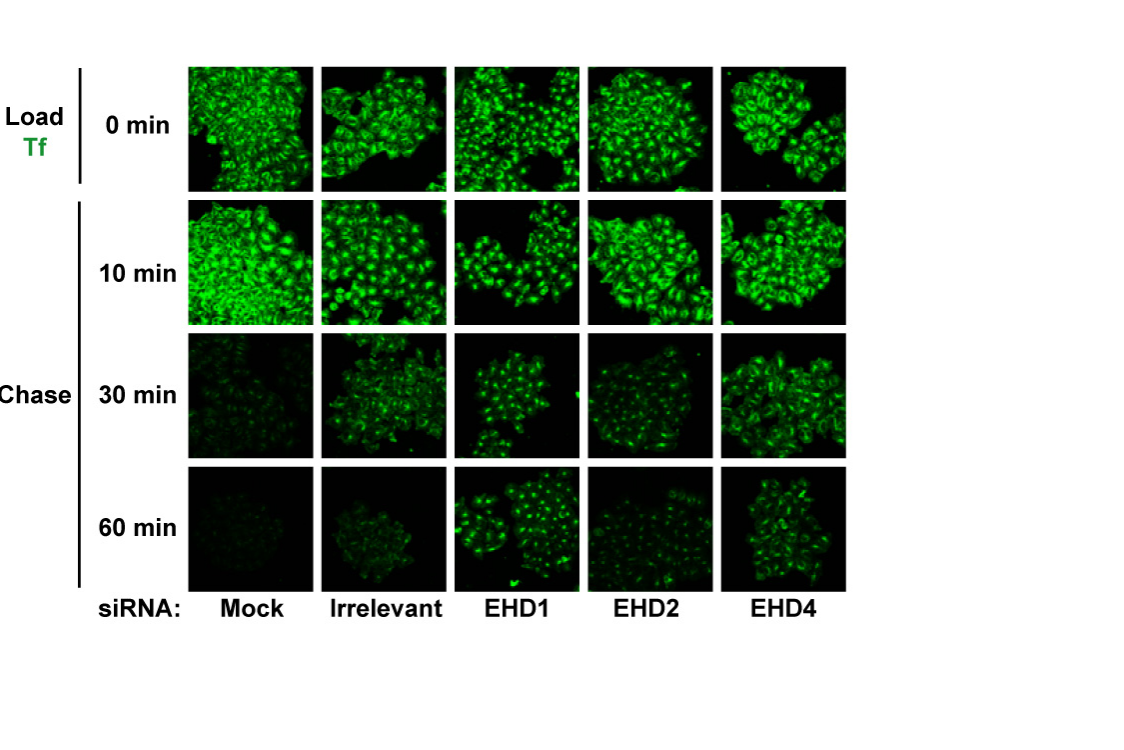
**siRNA-mediated EHD protein knock-down effects on transferrin recycling**. Following EHD protein knock-down, transferrin loading was carried out essentially as in Figure 10 except that Transferrin-coupled Alexa Fluor 488 was loaded for 30 min. Following transferrin loading, cells were washed with ice-cold PBS, changed to serum-containing media and allowed to recycle transferrin at 37°C for various time points. The cells were fixed and scanned using a confocal microscope equipped with a 40× objective lens. Knock-down cells retaining more transferrin than irrelevant controls at 60 min of chase were considered to be delayed in recycling transferrin. Irrelevant siRNA mildly affected transferrin recycling at 30 min of chase as compared to mock. Bar, 10 μm.

We also performed a transferrin recycling assay on these cells where labelled transferrin was loaded and chased for different time points. This assay allowed visualization of the compartment in which transferrin was trapped as it became demarcated during the course of the chase. Knock-down of EHD1 resulted in retention of considerable proportions of transferrin at 60 min of chase when compared to the irrelevant siRNA control (Figure 11). Cells with EHD4 knock-down retained less transferrin during the chase when compared to EHD1 knock-down yielding an intermediate phenotype while EHD2 knock-down had little effect as compared to the irrelevant siRNA control. Knock-down of EHD1 resulted in prolonged retention of transferrin in a perinuclear compartment while EHD4 knock-down led to retention in a peripheral compartment (Figure 11). The transferrin loading and recycling assays confirm that EHD1 and 2 function in the ERC to regulate transferrin recycling, with EHD1 being a dominant regulator, while EHD4 regulates transferrin exit out of the EE. The lack of an effect on transferrin recycling with EHD2 knock-down seen here is in agreement with our results using the EHD2 ΔEH construct suggesting that EHD2 functions at a location in the ERC where it exerts minimal effects on transferrin recycling. Irrelevant siRNA also seemed to affect transferrin recycling at 30 min but was comparable to untransfected cells at 60 min of chase. While it is not presently feasible to demonstrate concurrent knock-down of multiple EHD proteins in individual cells while showing the transferrin phenotype, generation of single and multiple EHD knock-out cells derived from mice with targeted deletions of EHD proteins will allow future analyses to address the authenticity of the phenotypes seen here.

## Discussion

To determine whether all EHD proteins had a functional role in endocytic trafficking, we took advantage of the availability of a *C. elegans *mutant in which the EHD ortholog RME-1 was non-functional. We found that all EHD proteins could rescue the basolateral trafficking defect in the intestine of mutant worms and thus demonstrate for the first time that all EHD proteins retain an ancestral endocytic recycling function (Figure 1). Analysis of multiple cell lines indicate that EHD1-4 proteins are co-expressed in several human cell lines concurrently (Figure 2). Moreover, EHD proteins were found to be ubiquitously expressed in the mouse tissues analyzed, albeit with tissue-specific differences in protein levels (Figure 3). Given that all four human EHD proteins function in our *C. elegans *rescue experiments, their co-expression in mammalian cells (our studies and others [16]) suggests that either mammalian EHD proteins play a functionally redundant role in endocytic recycling or that they have attained additional paralog-specific functions. As discussed below, our findings suggest that EHD proteins play both redundant and specific roles.

Yeast two-hybrid studies as well as co-expression in mammalian cells indicate that EHD1 and 3 form homo- and hetero-oligomers [15]; this process is mediated by a functional P-loop and coiled-coil domain, and is independent of the EH domain [9,16]. However, oligomerization of EHD2 and 4 have not been previously examined using co-IP analyses. In our analyses, we found that each EHD protein homo- and hetero-oligomerized, however, hetero-oligomerization between EHD proteins was variable. For example, EHD2 oligomerized weakly with EHD3 and 4 (Figure 4A). Consistent with observations that the EH domain is not required for oligomerization [9,15,16], the ΔEH mutants oligomerized similar to wild type EHD proteins (Figure 4B). Our results provide the first complete analysis of the oligomerization capabilities of EHD proteins in mammalian cells using a co-IP strategy. These results broadly agree with the predicted coiled-coil structure deduced for each protein (Additional File 3). Co-IP of endogenous EHD2 and 4 in mouse tissue lysates using anti-EHD1 antibodies supports the likelihood that oligomerization is physiologically relevant. Since EHD1 and 3 have been shown to bind to NPF-containing proteins in an oligomerization-dependent (e.g. Rab11FIP2) and -independent manner (e.g. Rabenosyn-5) [16], it is tempting to speculate that oligomerization might not only influence membrane binding but also the selection of protein interaction partners.

The results of our colocalization analyses in HeLa cells mirrored the co-IP results: EHD1, 3 and 4 substantially colocalized while EHD2 showed partial colocalization with EHD4 and little colocalization with EHD1 and 3 (Figure 6). While it is possible that the localization of individual EHD proteins might change upon co-overexpression of other EHD proteins, this appears relatively unlikely since EHD1, 3 and 4 did not appear to change their pattern of localization when expressed with each other; however, EHD4 structures did appear to change upon co-expression with EHD2. In further support of the differential endocytic localization of EHD proteins, we observed that EHD1, 3 and 4 partially colocalized with Rab11 while EHD2 showed minimal colocalization (Figure 7). Interestingly, even though EHD1, 3 and 4 colocalized with Rab11, dramatic perinuclear clustering of Rab11-GFP-positive vesicles only occurred upon overexpression of EHD1 ΔEH and 3 ΔEH mutants (not EHD2 ΔEH and 4 ΔEH) suggesting that EHD1 and 3 may function in the same recycling pathway upstream of Rab11 (Figure 8). We were unable to detect any direct association between EHD proteins and Rab11 upon co-IP analysis (data not shown) similar to a previous report [16]. Knock-down of EHD1 induced the perinuclear clustering of Rab11-GFP similar to that of overexpression of EHD1 ΔEH, however, we were unable to test whether this was the case with EHD3 knock-down due to the inefficiency of EHD3 knock-down (Additional File 6).

A recent study showed that EHD1 and 3 interact with Rab11-FIP2, a Rab11 interacting protein, via EH-NPF interactions, and recruit Rab11-FIP2 to EHD-containing membranes in a P-loop and coiled-coil domain-dependent manner; however, the authors were unable to show an interaction between Rab11 and either EHD1 or 3 [16]. Rab11-FIP2 binds to both the GDP- and GTP-bound forms of Rab11 and is thought to recruit Rab11 to the membrane [39,40]. We hypothesize that EHD1 and 3 ΔEH mutants might interfere with their wild type protein function since they might not bind to NPF motifs on Rab11-FIP2. Since oligomerization of EHD1 and 3 was shown to be required for Rab11-FIP2 binding [16], the lower propensity for EHD2 and 4 to hetero-oligomerize might spare Rab11-FIP2 function leading to the inability of EHD2 and 4 ΔEH mutants to induce Rab11 clustering. It will be of great interest to assess whether EHD2 and 4 interact with Rab11-FIP2.

Our analyses of the impact of EHD proteins on transferrin exit from the ERC revealed that overexpression of wild type EHD proteins retarded this process (Figure 9B, black). While paradoxical, this finding is not unprecedented, as overexpression of other proteins involved in endocytic traffic, such as Rififylin, the RING finger and FYVE-like domain ERC protein, also led to a block in transferrin recycling [33] perhaps due to disruption of functional protein complexes and/or sequestration of effector proteins. Overexpression of EHD1, 3 and 4 ΔEH mutants further increased the transferrin exit block while that of EHD2 ΔEH did not (Figure 9B, grey).

The role of EHD proteins in transferrin trafficking was further clarified by the use of siRNA-mediated EHD knock-down. Transferrin loading and recycling experiments in siRNA-transfected cells revealed that all EHD proteins regulate transferrin trafficking (Figure 10A–C, Figure 11), in agreement with the results of the dominant-negative mutant approach. These experiments together with published studies on EHD3 [16] allow the classification of EHD proteins into two groups based on their effects on transferrin: EHD1 and 2 appear to regulate exit of transferrin out of the ERC and hence are ERC regulators (Figure 10A–C, Figure 11) while EHD3 [16] and 4 function in the EE and hence are EE regulators. Our results in this regard confirm the studies of the Caplan group using EHD1 and 3 knock-down but extend these to EHD2 and 4.

**Figure 12:**
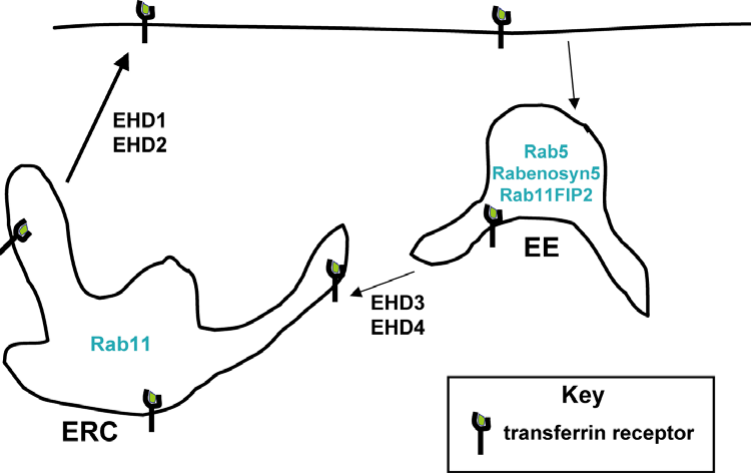
**Model of EHD-dependent and EHD-independent trafficking from the early and recycling endosomes**. Membrane-bound receptors and their cargo (such as the transferrin receptor and transferrin, respectively) that are destined for recycling to the cell surface following endocytosis can be internalized into the early endosome (EE) compartments, transferred to the endocytic recycling compartment (ERC) before returning to the cell surface. From siRNA-mediated depletion of EHD proteins and transferrin loading and recycling experiments here and elsewhere (Naslavsky *et al*. 2006) [16], EHD3 and 4 are hypothesized to regulate transferrin trafficking from the EE to the ERC while EHD1 and 2 regulate transferrin exit from the ERC. Proteins such as Rabenosyn5 and Rab11-FIP2 associate with EHD proteins through EH-NPF interactions and facilitate endosomal recycling.

Our biochemical and functional analyses of EHD proteins together with recent studies from other groups lead to a speculative model depicted in Figure 12. The four EHD proteins are differentially distributed within the recycling pathway with EHD3 and 4 functional in the EE and EHD1 and 2 in the ERC. At these locations, they could interact through their conserved EH domains with either similar or diverse NPF-containing partners. Distinct oligomers, formed as a result of preferential oligomerization through coiled-coils, could also mediate the recruitment of different EH domain binding partners in each compartment conferring a degree of selectivity to EHD function. For example, the presence of an apparently non-redundant EHD (EHD2) in the recycling endosome might help direct traffic into Rab11-dependent and -independent pathways and increase the versatility of recycling at the ERC. Further biochemical, cell biological and genetic studies on this conserved family of proteins will lead to an improved understanding of the endocytic recycling pathway.

## Conclusion

Overall, our biochemical and functional analyses of the human EHD proteins, members of a newly identified EH domain-containing protein family, indicate that they share an ancestral function of regulating endocytic recycling. Studies using dominant negative mutants and siRNA unravelled some important differences between the members, EHD1 and 2 regulate recycling at the ERC while EHD3 and 4 regulate EE to ERC transport. Overall, EHD2 appears to be the most divergent member with respect to localization and functions that we tested, while EHD4 seemed to be intermediate between EHD1/3 and EHD2. As mentioned previously, EHD2 and 4 have been predominantly studied in specific cell types such as adipocytes and pheochromocytoma cells, respectively. However, all four EHD proteins have not been compared concurrently in a single cell type. The contrast between the common ancestral endocytic trafficking function indicated by the *C. elegans *experiments and differences among EHD proteins revealed by studies in HeLa cells suggest that EHD proteins have functionally diversified during their recent evolution to match the versatility demanded by the complexity of endocytic traffic in mammalian cells. Further, comparative biochemical and cell biological studies of the EHD protein family between mammalian and non-mammalian systems should, therefore, facilitate our understanding of these conserved endocytic regulators, as well as provide new insights into the functional diversity of mammalian endocytic compartments. EHD1 knock-out mice have been generated recently and do not display any abnormality other than slower transferrin recycling in isolated mouse embryonic fibroblasts; possibly, indicating the overall redundancy of the EHD protein family [41]. Availability of single and multiple knock-out mice should further help to delineate EHD function along various steps in the recycling pathway.

## Methods

### Expression constructs

Sequences encoding human EHD proteins were PCR amplified from clones (ImageClone 5229002 for EHD1, 4908085 for EHD2, 5459130 for EHD3 (ATCC) and Ultimate ORF clone IOH6327 for EHD4 (Invitrogen)) using the primers listed in Additional File 7. The PCR products were cloned into pENTR/SD/D-TOPO vector following the manufacturer's instructions (Invitrogen) and sequences of correct clones were verified. The inserts were then transferred into pcDNA-pDEST47 (Invitrogen) vector using an LR Clonase reaction for CMV promoter-driven expression as C-terminal GFP-fusion proteins. GFP-tagged ΔEH deletion mutants were generated using reverse PCR primers excluding the C-terminal EH domain, the sizes of the mutant proteins with respect to number of amino acids were: EHD1 ΔEH (1–435), EHD2 ΔEH (1–439), EHD3 ΔEH (1–434) and EHD4 ΔEH (1–437); these mutants also contain an N-terminal Myc-tag. Myc-tagged wild type and ΔEH deletion mutants were also generated using the PCR with the inclusion of sequences for the Myc-tag in the forward primers followed by LR-mediated transfer into pcDNA-6.2/cLumio-DEST vector. While Myc-tagged proteins proved very useful in biochemical analyses, anti-Myc immunostaining was not fully comparable to corresponding EHD-GFP fluorescence patterns. To independently confirm the EHD-GFP localization patterns, DsRed-Monomer-tagged wild type EHD constructs were generated specifically for use in colocalization studies using forward primers with an Xho I site and reverse primers with a Hind III site, and cloned into Xho I-Hind III cut DsRed-Monomer-N1 vector (Clontech). All DsRed constructs used in this study were DsRed-Monomer and will be referred to as DsRed.

The EHD3 ImageClone was derived from a neuroblastoma and was found to lack a cytosine (C) at position 1552 of the ORF predicted by the Unigene sequence (ACCESSION: NM_014600), which we confirmed using the reverse transcription-PCR cloning and sequencing of EHD3 mRNA from 20 separate clones from four fibroblast and mammary epithelial cell lines. Therefore, the missing C in the ImageClone-derived EHD3 was replaced by site-directed mutagenesis. Primers used in the study are listed in Additional File 7.

The Rab5-GFP and Rab11-GFP constructs in EGFPN1 vector were provided by Dr. Victor Hsu (Brigham and Women's Hospital, Harvard Medical School, Boston, MA). The GFP-MTMR3 construct was provided by Dr. Michael Clague (University of Liverpool, Liverpool, UK). The Myc-SNX1 construct was provided by Dr. JoAnn Trejo (UNC School of Medicine, Chapel Hill, North Carolina).

### Cell culture

HeLa cells (obtained from Dr. Victor Hsu) and human embryonic kidney (HEK) 293T cells were grown in Dulbecco's Modified Eagle Medium (DMEM) containing 5% fetal bovine serum (FBS, Hyclone Inc., Logan, UT), 20 mM HEPES, pH 7.35, 1 mM sodium pyruvate, 1 mM each of nonessential amino acids, 100 units/ml penicillin and 100 μg/ml streptomycin (all supplements from Invitrogen).

Primary (76N) and immortalized (16A5, MCF10A) human mammary epithelial cell strains were grown in DFCI-1 medium as described [42]. Human Breast carcinoma cell line T47D, cervical carcinoma cell line SiHa and osteosarcoma cell line U2OS were cultured in alpha-minimal essential medium (Invitrogen) supplemented with 5% fetal calf serum (Hyclone Inc., Logan, UT).

### Gene knock-down by small interfering RNA (siRNA)

Small interfering RNA (siRNA) oligonucleotides (synthesized by Dharmacon, Lafayette, CO) were transfected using Oligofectamine (Invitrogen) following the manufacturer's instructions. Demonstrable knock-down of protein expression was seen 48 h after transfection. siRNA sequences targeting EHD1 and EHD3 were as in Caplan *et al*. 2005 (EHD1, 5'-gaa aga gat gcc caa tgt c, bases 945–963; EHD3, 5'-act gga cat ctc tga tga g, bases 945–963) [16]. siRNA sequences targeting EHD2 and EHD4 (EHD2, 5'-gtc tac atc ggc tcc ttc t, bases 754–772; EHD4, 5'-tgg agg acg ccg act tcg a, bases 158–176) were generated using the SFold algorithm [43]. An irrelevant siRNA control (siCONTROL Non-Targeting siRNA, Cat. #D-001210-01-20) was purchased from Dharmacon (Lafayette, CO).

### Antibodies and Western blotting

Rabbit polyclonal antibodies were raised against synthetic peptides coupled through an N-terminal cysteine to Keyhole Limpet Hemocyanin (KLH) (CVSKDARRKKEPELF for EHD1, CSKRRHKGSAE for EHD2, CNLKRMQDQLQAQ for EHD3, and CSHRKSLPKAD for EHD4) using a commercial vendor (Animal Pharm Services, Inc., Healdsburg, CA). Primary immunization with 150 μg of KLH-peptide in Freund's Complete Adjuvant was followed by three booster injections of 100 μg in Freund's Incomplete Adjuvant. The monoclonal antibody 9E10 (anti-Myc) [44] was purified from serum-free culture supernatants using Protein G Sepharose 4 Fast Flow beads (Amersham Biosciences, Piscataway, NJ). Rabbit anti-green fluorescent protein (anti-GFP, sc-8334) was from Santa Cruz Biotechnology (Santa Cruz, CA).

For Western blotting, cell lysates were prepared in Laemmli SDS-PAGE sample buffer, and protein concentration was determined using the Bio-Rad D_c _Protein Assay (Bio-Rad Laboratories, Hercules, CA) with bovine serum albumin as standard. Aliquots of 100 μg protein lysate were separated by SDS-PAGE, transferred to polyvinylidene difluoride (PVDF) membranes (PerkinElmer, Boston, MA) and immunoblotted with 1:2000 dilutions of the indicated antisera, followed by 1:10,000 dilution of horseradish peroxidase (HRP)-conjugated protein A (Cappel/Organon Teknika Corp., West Chester, PA), as described [45]. Signals were detected using Western Lightning Chemiluminescence Reagent Plus (PerkinElmer, Boston, MA) and Kodak X-Omat Blue XB-1 film (PerkinElmer, Boston, MA). Figures were prepared by direct scanning of films with a Hewlett Packard Scanjet 7400c scanner and Photoshop 6.0 software.

### Preparation of mouse tissue lysates

Mice (18 week-old male and female C57BL/6J) were sacrificed and organs were dissected (brain, heart, lung liver, spleen, kidney, intestine, and testes (male) or mammary gland (female)), washed thoroughly in PBS, chopped into fine pieces, and rocked at 4°C in tissue lysis buffer (50 mM Tris-HCl (pH7.5), 150 mM NaCl, 1 mM EDTA, 2.5 mM EGTA, 1 mM DTT, 0.1% Tween-20, 10% glycerol, 2 mM Na_3_VO_4_, 20 mM NaF, 1 mM PMSF) overnight. 100 μg of aliquots of lysate protein were separated using 8% SDS-PAGE and Western blotted as described above.

### Co-immunoprecipitations

HEK 293T cells were transfected with 2.5 μg each of Myc-EHD and EHD-GFP DNA constructs using a modified version of the calcium phosphate method [46], grown for 26–30 h, lysed with cold lysis buffer (1% Triton X-100, 150 mM NaCl, 50 mM Tris-HCl, pH 7.6) supplemented with 0.1 mM phenylmethylsulfonyl fluoride, 1 mM sodium orthovanadate, and 10 mM sodium fluoride [47], and rocked at 4°C overnight or 1–2 hours for Myc-EHD and Myc-EHD ΔEH immunoprecipitations, respectively. Immunoprecipitations were carried out as described previously [48,49] using 1 mg aliquots of protein lysate, 3 μg of anti-Myc antibody and 20 μl of Protein G Sepharose beads. Beads were washed five times, and bound proteins were eluted in Laemmli sample buffer with 2-mercaptoethanol and resolved by 8% SDS-PAGE followed by immunoblotting. Myc-tagged proteins were detected with mouse-anti-Myc 9E10 at 500 ng/ml and rabbit anti-mouse IgG (H+L)-HRP conjugate (Zymed, San Fransisco, CA) at 1:25,000. GFP-tagged proteins were detected with rabbit anti-GFP (Santa Cruz Biotechnology, Santa Cruz, CA) at 200 ng/ml followed by Protein A-HRP (Zymed, San Fransisco, CA) at 1:25,000. Chemiluminescence detection was as described above.

### Transfection, immunofluorescence and confocal microscopy

HeLa cells were grown on 12-mm diameter glass coverslips (Fisher Scientific, Pittsburgh, PA) for 1 day, transfected using the calcium phosphate co-precipitation method with the desired plasmids for 24 h, rinsed with phosphate-buffered saline (PBS) and fixed with 4% paraformaldehyde in PBS at 4°C o Cvernight. The cells were rinsed with PBS, followed by the addition of permeabilization and blocking buffer (PBS containing 5% FBS and 0.05% saponin from Quillaja bark (Sigma, St. Louis, MO) at 25°C for 30 min. For anti-Myc staining, cells were incubated with mouse-anti-Myc (0.5 μg/ml) at 25°C for 1 h. After rinsing with wash buffer (0.05% saponin in PBS), cells were incubated with Alexa Fluor 594-conjugated goat anti-mouse IgG (Molecular Probes, Eugene, OR, A11005) at 25°C for 1 h. The cells were washed extensively with wash buffer and mounted on glass slides using Vectasheild with DAPI for nuclear staining (Vector Laboratories, Burlingame, CA). Fluorescently-stained cells were scanned using a Nikon Eclipse 80i confocal microscope equipped with a Nikon D-eclipse C1 scanning head (Nikon) and analyzed using the EZ-C12.10 software.

### Live cell imaging

Live cell video microscopy was performed on transfected HeLa cells grown on Poly-D-Lysine-coated 35-mm coverslip bottom dishes (BD Biocoat Cell Environments). During imaging, cells were immersed in CO_2_-independent medium (Life Technologies, Grand Island, NY). The cells were imaged every 5 seconds for 7 minutes using a 100× oil immersion objective on an inverted microscope (Model TE2000-U; Nikon) equipped with a charge-coupled device camera controlled by Metamorph software (Universal Imaging Corp.). Image control and post-capture image analysis were performed using MetaMorph software.

### Transferrin recycling

Wild type or Myc-EHD ΔEH-transfected HeLa cells were loaded with Alexa Fluor 488-labeled transferrin (Molecular Probes, Eugene, OR, T-13342) at 10 μg/ml in internalization media (DMEM, 10 mM HEPES pH 7.4, 0.1% BSA) at 37°C for 30 min, rinsed with ice-cold PBS followed by transferrin chase by incubation at 37°C in regular serum-containing media [38]. At the indicated time points, cells were washed twice and fixed with 4% paraformaldehyde, stained with anti-Myc antibody and mounted as above, followed by image acquisition. For colocalization of transferrin with Rab5 and Rab11 in siRNA-transfected cells, cells were transfected with Rab5 and Rab11-GFP 24 h after siRNA transfection using the FuGene 6 reagent (Roche Diagnostics, Indianapolis, IN). After 24 hours, the cells were loaded with transferrin as described above, fixed in 4% paraformaldehyde and mounted on glass slides using Vectasheild with DAPI for nuclear staining (Vector Laboratories, Burlingame, CA). Confocal analyses were performed with Zeiss inverted LSM510 confocal microscopy system.

### *C. elegans *strains, culture conditions, and *rme-1*(*b1045*) rescue experiments

C. elegans worms were cultured at 22°C under standard growth conditions [50]. Strains used in this study were: Bristol strain N2 (wild type) and *rme-1*(*b1045*) with a mutation in the EHD ortholog [10]. To test whether human EHD proteins could rescue the vacuolated intestine phenotype of rme-1 worms, full length human EHD cDNAs were expressed downstream of a worm intestine-specific Vha-6 promoter [30] in pENtr vector containing the SL2-gfp operon cassette [51] (details of plasmids available upon request). The rescue constructs (50 ng/μl) were co-injected with a GFP marker (myo::gfp at 100 ng/μl) into the gonads of hermaphrodite *rme-1*(*b1045*) worms using standard methodology [52]. The intestinal vacuoles were counted in three independent lines of transgenic adult worms (grown 3–4 days) expressing GFP in intestinal cells. At least 25 worms were counted for each independent line. The basolateral endocytosis assay of the intestinal vacuoles was performed in adult hermaphrodites by microinjection of 1 mg/mL Texas-Red BSA into the pseudocoelom as described [10].

## Abbreviations

^1^The abbreviations used are: co-immunoprecipitation = co-IP, EE = early endosome, Eps15 = epidermal growth factor receptor pathway substrate 15, EH = Eps15 Homology, EHD protein = EH domain-containing protein, ERC = endocytic recycling compartment, NPF = Asn-Pro-Phe, RME-1 = receptor-mediated endocytosis-1, P-loop = phosphate-binding loop, Rab11-FIP2 = Rab11-Family Interacting Protein 2, Tf = transferrin, TR-BSA = Texas Red-BSA, siRNA = small interfering RNA.

## Authors' contributions

MG designed and carried out the cloning, colocalization, transferrin loading and recycling experiments, siRNA knock-downs and drafted the manuscript. GY designed and made expression clones, analyzed cell lysates, and helped with drafting the manuscript. MAR carried out co-immunoprecipitation experiments using cell lysates and mouse extracts, designed and tested knock-down constructs and helped with drafting the manuscript. AS performed the *C. elegans *rescue experiments. PTP helped with Western blotting. QG helped with acquisition of data and provided critical comments during the study and critically reviewed the manuscript. VB provided critical comments and helped with the design and coordination of the study. HB conceived of the study and participated in its design and coordination and helped to draft the manuscript. All authors read and approved the final manuscript.

## Supplementary Material

Additional File 1 **Determination of the specificity of EHD peptide antisera**. HEK 293FT cells in 100-mm tissue culture dishes were transiently transfected with DNA encoding a single EHD-GFP (6 μg) construct. Cell lysates were prepared as in Methods. Aliquots of 100 μg were loaded onto an 8% SDS-PAGE gel, transferred to a PVDF membrane, and immunoblotted with specific EHD anti-sera as shown. Relative molecular weight (MW) markers are indicated in kiloDaltons (kD). Click here for file

Additional File 2 **Western blot of whole cell lysates of GFP-tagged EHD proteins used in **Figure 4. Aliquots of 100 μg of the lysates used for co-immunoprecipitations (co-IP) in Figure 4 were run on the same gel as those in Figure 4, transferred to PVDF membranes, and immunoblotted in parallel with anti-GFP antibodies. (A) Whole cell lysates for Figure 4A. (B) Whole cell lysates for Figure 4B. (C) Control IPs using 1 mg of whole cell lysates (WCL) and co-IPs were carried out as described in Methods using GFP-myotubularian-related protein 3 (MTMR3), Myc-sorting nexin 1 (SNX1), Myc-EHD1 and EHD1-GFP. Lanes 1–3: WCL, 100 μg. Lanes 4–6: 1 mg IP. Relative molecular weight (MW) markers are indicated in kiloDaltons (kD). The heavy chain of the mouse IgG (IgH) is also shown indicating similar levels of antibody (anti-Myc, 9e10) were used for the IP. Click here for file

Additional File 3 **Coiled-coil prediction plots of EHD proteins using COILS**. Primary amino acid sequences of EHD1-4 were subjected to analysis using the COILS program [53] to predict the probability of the protein to adopt a coiled-coil conformation using a 28 residue scan. Click here for file

Additional File 4 **Time-lapse movie of a HeLa cell co-transfected with Rab11-GFP and EHD1-DsRed**. GFP-tagged Rab11 (green) and DsRed-tagged EHD1 (red) were co-transfected into HeLa cells plated on autoclaved glass coverslips. Movie images were captured as described in Methods. Green and red vesicles are seen to move towards each other and transiently merge (yellow). Click here for file

Additional File 5 **Effect of overexpression of EHD2-4 wild type and ΔEH mutants on transferrin exit from the ERC**. Methodology as described in Figure 9. Click here for file

Additional File 6 **siRNA Western Blot**. (A) Lysates were prepared as described in Methods and 100 μg were loaded onto a 10% SDS-PAGE gel, transferred to a PVDF membrane, and immunoblotted with specific EHD anti-sera as shown. Relative molecular weight (MW) markers are indicated in kiloDaltons (kD). (B) The percentage (%) of remaining EHD proteins after siRNA treatment was calculated by normalizing the intensity of the EHD band with respect to the loading control and comparing it with the bands in the control siRNA-treated lanes. Click here for file

Additional File 7 **List of primers used to PCR-amplify EHD genes**. Sequences corresponding to the gene are in uppercase. Sequences corresponding to the Myc-tag are italicized. Restriction enzyme sites are underlined. A "CACC" sequence was included in the forward primers for TOPO-cloning into entry vectors. Click here for file
