# Supplementary figures and images for: Shared as well as distinct roles of EHD proteins revealed by biochemical and functional comparisons in mammalian cells and C. elegans
Source: BMC Cell Biol. 2007 Jan 18;8:3. doi: 10.1186/1471-2121-8-3 (PMC1793994; doi:10.1186/1471-2121-8-3)

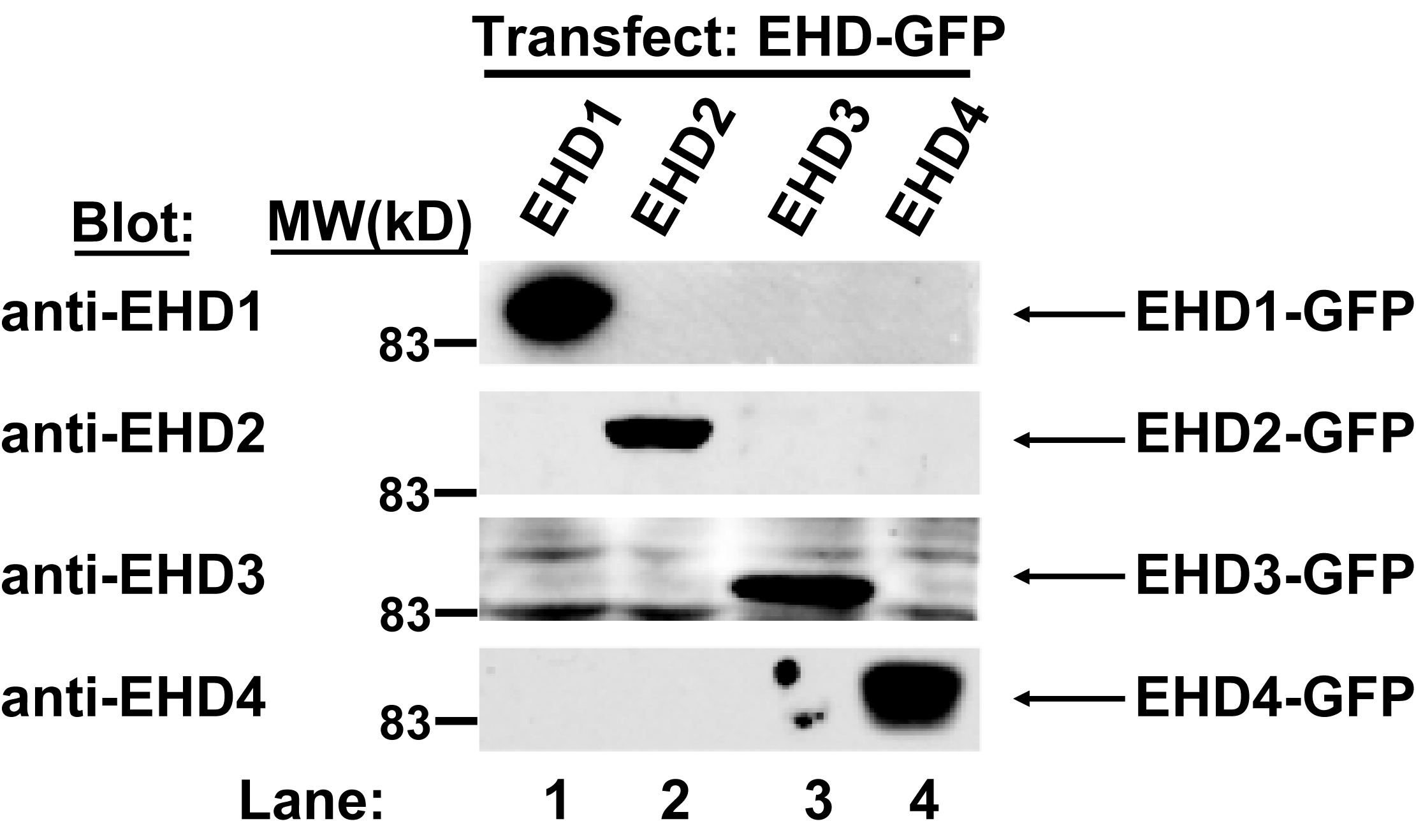

Supplement: Additional File 1 — Determination of the specificity of EHD peptide antisera. HEK 293FT cells in 100-mm tissue culture dishes were transiently transfected with DNA encoding a single EHD-GFP (6 μg) construct. Cell lysates were prepared as in Methods. Aliquots of 100 μg were loaded onto an 8% SDS-PAGE gel, transferred to a PVDF membrane, and immunoblotted with specific EHD anti-sera as shown. Relative molecular weight (MW) markers are indicated in kiloDaltons (kD). [file 1471-2121-8-3-S1.tiff]

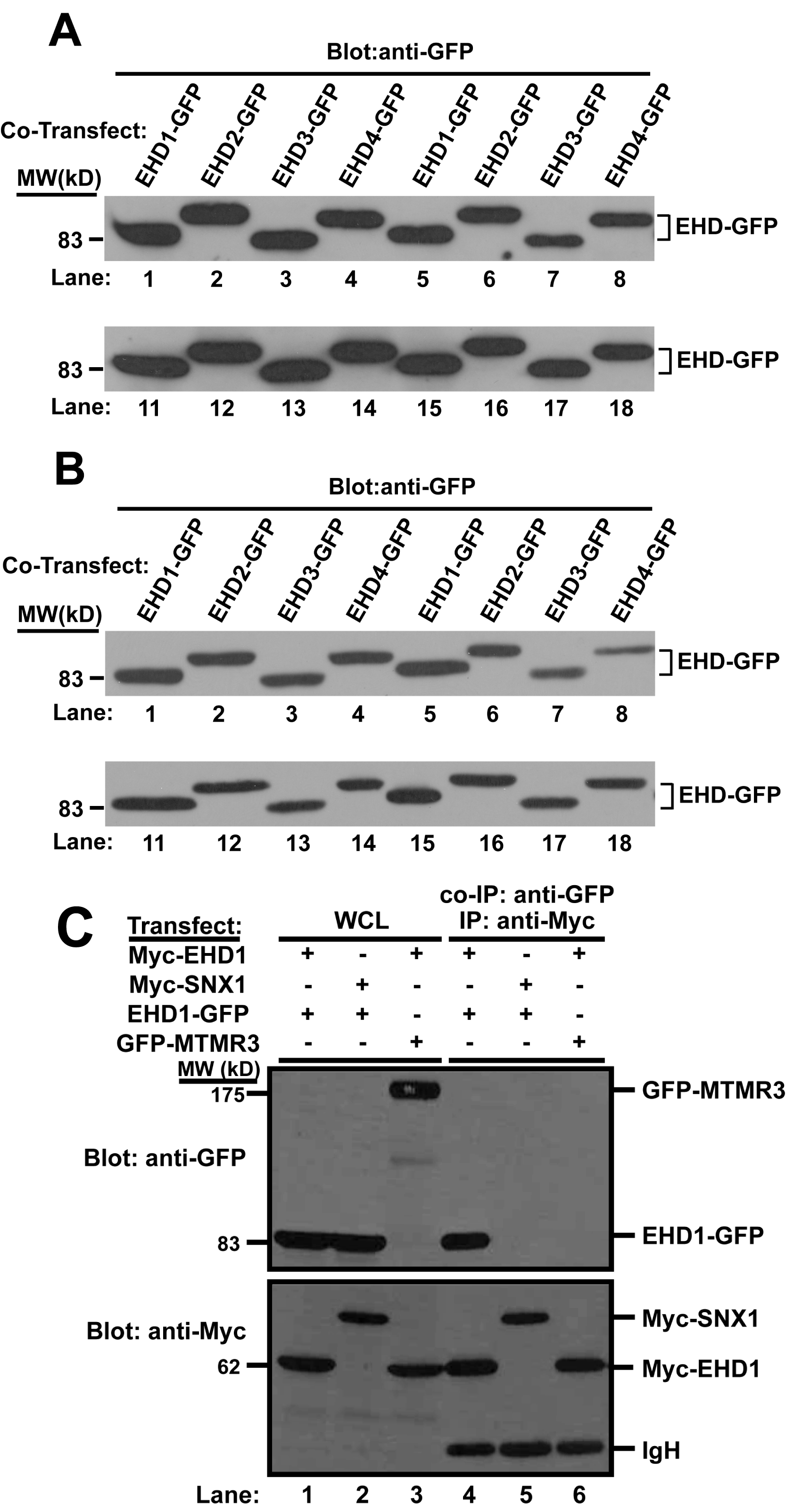

Supplement: Additional File 2 — Western blot of whole cell lysates of GFP-tagged EHD proteins used in Figure 4. Aliquots of 100 μg of the lysates used for co-immunoprecipitations (co-IP) in Figure 4 were run on the same gel as those in Figure 4, transferred to PVDF membranes, and immunoblotted in parallel with anti-GFP antibodies. (A) Whole cell lysates for Figure 4A. (B) Whole cell lysates for Figure 4B. (C) Control IPs using 1 mg of whole cell lysates (WCL) and co-IPs were carried out as described in Methods using GFP-myotubularian-related protein 3 (MTMR3), Myc-sorting nexin 1 (SNX1), Myc-EHD1 and EHD1-GFP. Lanes 1–3: WCL, 100 μg. Lanes 4–6: 1 mg IP. Relative molecular weight (MW) markers are indicated in kiloDaltons (kD). The heavy chain of the mouse IgG (IgH) is also shown indicating similar levels of antibody (anti-Myc, 9e10) were used for the IP. [file 1471-2121-8-3-S2.tiff]

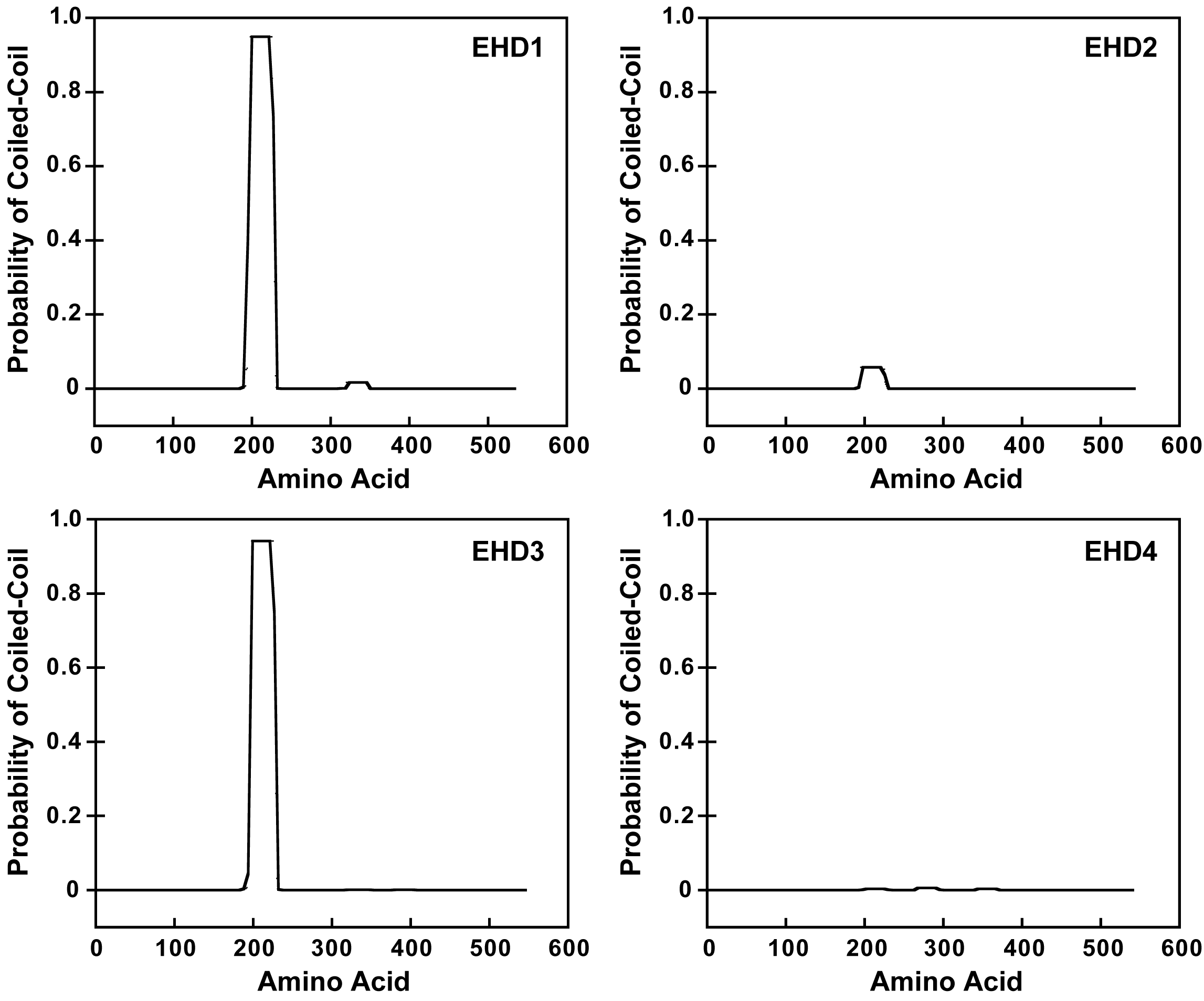

Supplement: Additional File 3 — Coiled-coil prediction plots of EHD proteins using COILS. Primary amino acid sequences of EHD1-4 were subjected to analysis using the COILS program [53] to predict the probability of the protein to adopt a coiled-coil conformation using a 28 residue scan. [file 1471-2121-8-3-S3.tiff]

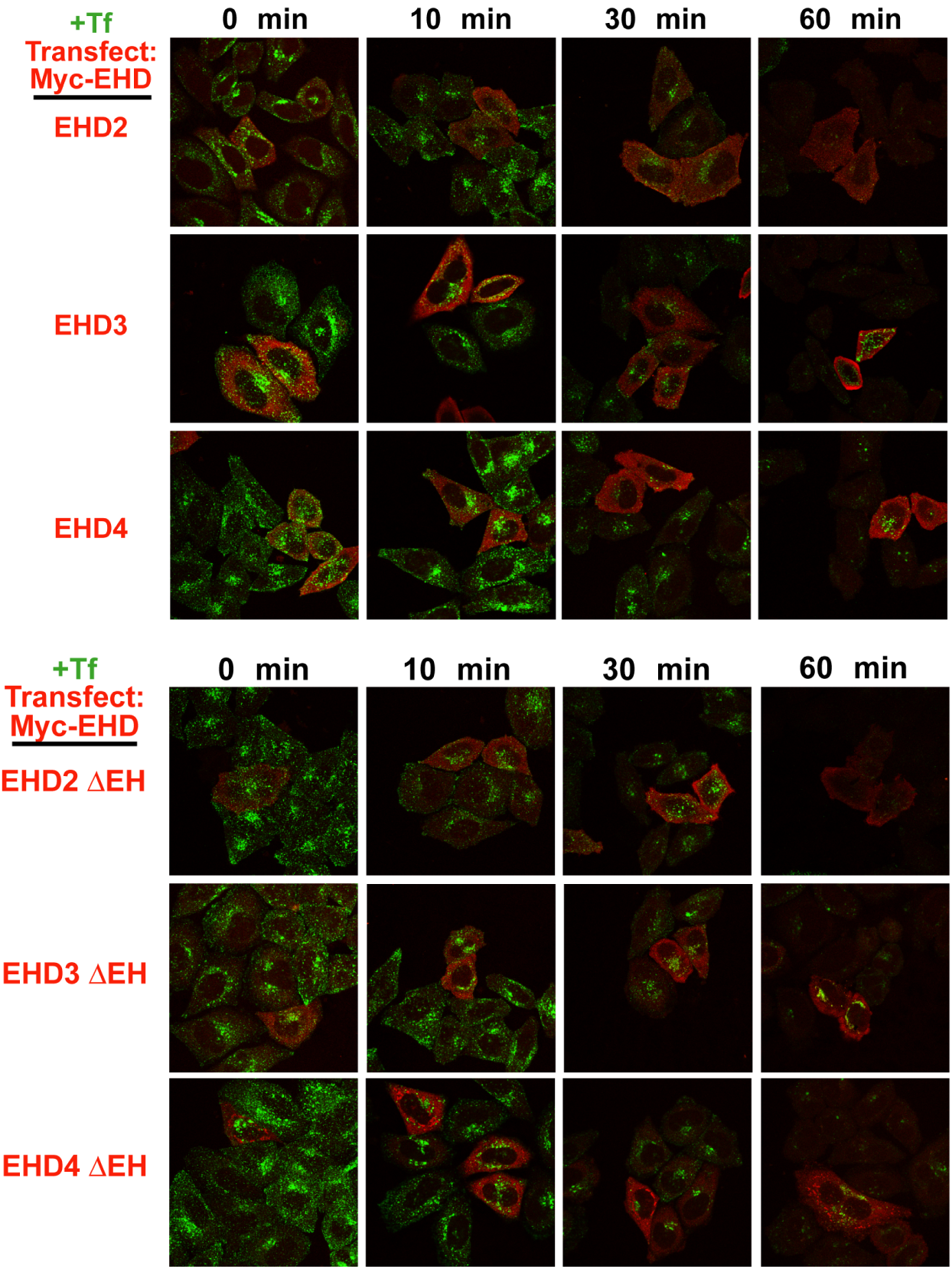

Supplement: Additional File 5 — Effect of overexpression of EHD2-4 wild type and ΔEH mutants on transferrin exit from the ERC. Methodology as described in Figure 9. [file 1471-2121-8-3-S5.tiff]

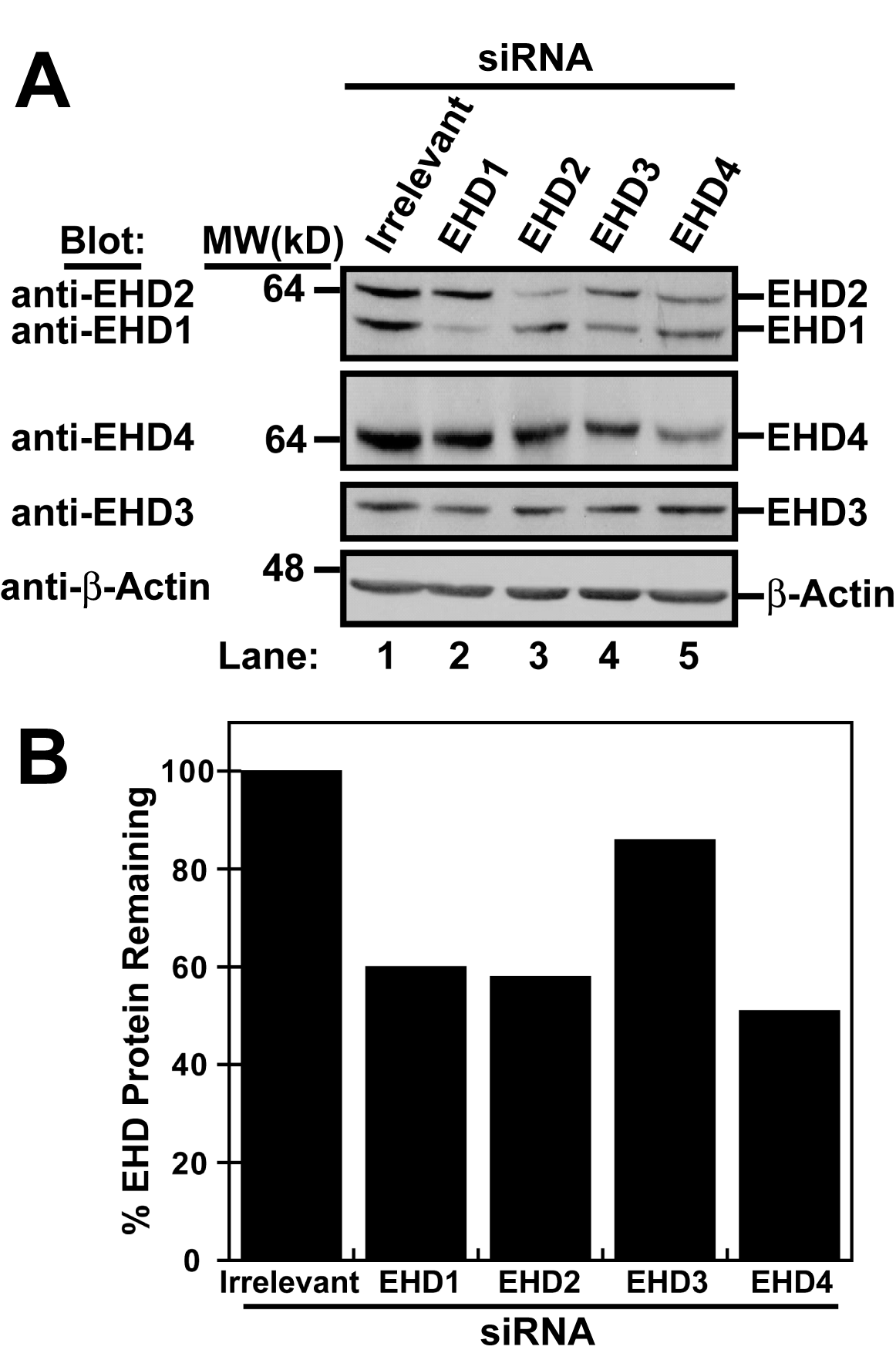

Supplement: Additional File 6 — siRNA Western Blot. (A) Lysates were prepared as described in Methods and 100 μg were loaded onto a 10% SDS-PAGE gel, transferred to a PVDF membrane, and immunoblotted with specific EHD anti-sera as shown. Relative molecular weight (MW) markers are indicated in kiloDaltons (kD). (B) The percentage (%) of remaining EHD proteins after siRNA treatment was calculated by normalizing the intensity of the EHD band with respect to the loading control and comparing it with the bands in the control siRNA-treated lanes. [file 1471-2121-8-3-S6.TIFF]
