# Supplementary material for: Shared as well as distinct roles of EHD proteins revealed by biochemical and functional comparisons in mammalian cells and C. elegans
Source: BMC Cell Biol. 2007 Jan 18;8:3. doi: 10.1186/1471-2121-8-3 (PMC1793994; doi:10.1186/1471-2121-8-3)
Supplement: Additional File 7 — List of primers used to PCR-amplify EHD genes. Sequences corresponding to the gene are in uppercase. Sequences corresponding to the Myc-tag are italicized. Restriction enzyme sites are underlined. A "CACC" sequence was included in the forward primers for TOPO-cloning into entry vectors. [file 1471-2121-8-3-S7.doc]

Additional File 7- List of primers used to PCR-amplify EHD genes.

| **Primer** | **Sequence** | **Comments** |
| --- | --- | --- |
| EHD1F | caccATGTTCAGCTGGGTCAGCAAG | For cloning into entry vector |
| EHD1-1602R | CTCATGTCTGCGCTTGGAGG | For cloning into entry vector |
| EHD2 F | caccATGTTCAGCTGGCTGAAGCG | For cloning into entry vector |
| EHD2-1629R | CTCGGCGGAGCCCTTGTGG | For cloning into entry vector |
| EHD3F | caccATGTTCAGCTGGCTGGGTAC | For cloning into entry vector |
| EHD3-1605R | CTCGGCAACTTTCCTCTTGG | For cloning into entry vector |
| EHD4F | caccATGTTCAGCTGGATGGGGCG | For cloning into entry vector |
| EHD4-1623R | GTCGGCCTTGGGCAGGGAC | For cloning into entry vector |
| EHD3CF | GGGGCACGAGCTGCCCAACGAGCTGCCTGCC | For C insertion |
| EHD3CR | GGCAGGCAGCTCGTTGGGCAGCTCGTGCCCC | For C insertion |
| EHD1MycF | cacc*atggaacaaaaactcatctcagaagaggatctg*ATGTTCAGCTGGGTCAGC | For making Myc tagged EHD1 |
| EHD2MycF | cacc*atggaacaaaaactcatctcagaagaggatctg*ATGTTCAGCTGGCTGAAG | For making Myc tagged EHD2 |
| EHD3MycF | cacc*atggaacaaaaactcatctcagaagaggatctg*ATGTTCAGCTGGCTGGGT | For making Myc tagged EHD3 |
| EHD4MycF | cacc*atggaacaaaaactcatctcagaagaggatctg*ATGTTCAGCTGGATGGGG | For making Myc tagged EHD4 |
| dEHD1R | GATGCCCTCGCCGGCCCCCTCGCC | For making EHD1 - ΔEH |
| dEHD2R | GTCCGAGCCCTCCTCGCCGTCCTC | For making EHD2 - ΔEH |
| dEHD3R | GATACCTTCTCCAGCCCCCTCCCC | For making EHD3 - ΔEH |
| dEHD4R | GGCGCCCTCCTTGGCACCCTCCCC | For making EHD4 - ΔEH |
| EHDXhoF | aaggagctcgagACCATGTTCAGCTGG | For making DSred Monomer EHDs |
| EHD1HindIIIR | gcgcccaagcttCTCATGTCTGCGCTTGGA | For making DSred Monomer EHD1 |
| EHD2HindIIIR | gcgcccaagcttCTCGGCGGAGCCCTTGTG | For making DSred Monomer EHD2 |
| EHD3HindIIIR | gcgcccaagcttCTCGGCAACTTTCCTCTT | For making DSred Monomer EHD3 |
| EHD4HindIIIR | gcgcccaagcttGTCGGCCTTGGGCAGGGA | For making DSred Monomer EHD4 |
